# Supplementary material for: Policy assessments for the carbon emission flows and sustainability of Bitcoin blockchain operation in China
Source: Nat Commun. 2021 Apr 6;12:1938. doi: 10.1038/s41467-021-22256-3 (PMC8024295; doi:10.1038/s41467-021-22256-3)
Supplement: Supplementary file 1 — Supplementary Information [file 41467_2021_22256_MOESM1_ESM.pdf]

Supplementary Information for

Policy assessments for the carbon emission flows and sustainability of Bitcoin blockchain operation in China

Jiang et al.

Supplementary Tables

Supplementary Table 1 Variable descriptions

Type	Parameter	Definition	Unit	Source
Level	Miner cumulative Profits	Total accumulated profits of Bitcoin miner in China	USD	-
	GDP	Gross productivity of Bitcoin blockchain in China	USD	-
	Total Carbon Emission	Accumulated carbon emission of Bitcoin blockchain in China	kg	-
Rate	Miner profit rate	Bitcoin miners' income per month	USD/month	-
	Investment intensity	Investment intensity of Bitcoin miners	-	Küfeoğlu & Özkuran[1]; CBECI
	GDP growth	Gross domestic product added per month	USD/month	-
	Carbon emission flow	Carbon emission of Bitcoin blockchain per month	Kg/month	-
Auxil iary	Mining hash rate	Mining hashes per second of Bitcoin network	Trillion hashes/ second	BTC.com
	Mining efficiency	Average mining efficiency of Bitcoin network	Joule/ Trillion hashes	Küfeoğlu & Özkuran[1]; CBECI
	Mining power	Average mining power of Bitcoin network	Watt	-
	Network energy consumption	Monthly energy consumption of Bitcoin operations	Kilowatt hour	-
	Market access standard for efficiency	Market access standards for Bitcoin miners' efficiency	100%	-
	Power usage effectiveness	Energy usage effectiveness of Bitcoin mining centers	-	Stoll et al.[2]
	Coal-based energy consumption	Energy consumed by Bitcoin blockchain in Coal-based region	Kilowatt hour	-

Hydro-based energy consumption	Energy consumed by Bitcoin blockchain in hydro-rich region	Kilowatt hour	-
Coal-based energy carbon emission	Carbon dioxide generated by Coal-based region miners in Bitcoin blockchain	Kg	-
Hydro-based carbon emission	Carbon dioxide generated by Hydro-based region miners in Bitcoin blockchain	Kg	-
Carbon intensity of Coal-based energy	Emission factor of Coal-based energy in China	Kg/Kilowatt hour	Cheng et al.[3]
Carbon intensity of Hydro-based energy	Emission factor of Hydro-based energy in China	Kg/Kilowatt hour	Cheng et al.[3]
Miner site selection	proportions of Bitcoin server located in coal-based region	%	BTC.com
Carbon emission cost	Monthly carbon emission cost in Bitcoin blockchain	USD	-
Energy price	Average energy (electricity) price in China	USD/kwh	World Bank
Energy cost	Monthly energy (electricity) cost in Bitcoin blockchain	USD	-
Total mining operating cost	Sum of carbon cost and energy cost	USD	-
Carbon tax	Average taxation for industrial carbon emission	USD/Kg	World Bank
Block hash difficulty	Global block hash difficulty in Bitcoin blockchain	T	-
New block	New block generated by miners per month	-	-
Proportion of Chinese miners	The proportion of Chinese miners in global Bitcoin mining system	%	BTC.com; Küfeoğlu & Özkuran[1]
Block size	Bitcoin blockchain size per month	Megabyte	BTC.com
Transaction fee	Transaction fee per month	Bitcoin	BTC.com

Bitcoin Price	Periodical Bitcoin price	USD	-
Block reward	Monthly Bitcoin mined	Bitcoin	-
Mining Reward Halving mechanism	The mining reward Halving mechanism of Bitcoin	-	-

Supplementary Table 2 Initial value of auxiliary parameters in the BBCE model

Parameter	Value	Unit	Parameter	Value	Unit
Carbon tax	0.01	USD/kg	Market access standard for efficiency	100	%
Carbon intensity of coal-based energy	0.9	Kg/kwh	Power usage effectiveness	1.1	-
Carbon intensity of hydro-based energy	0.2	Kg/kwh	Miner site selection	40	%
Energy price	0.05	USD/kwh	Proportion of Chinese miners	70	%

Supplementary Figures

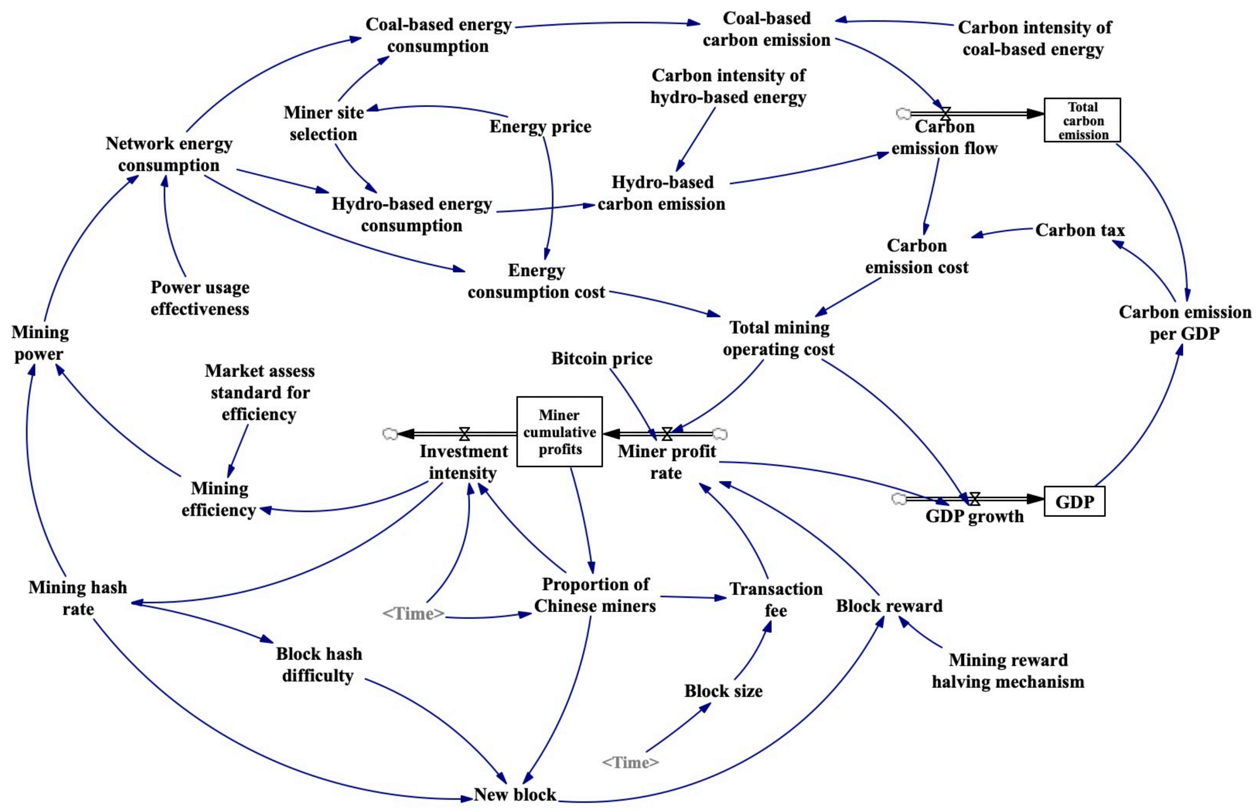

Supplementary Fig. 1 | Flow diagram of BBCE modelling. Parameters of the Bitcoin blockchain carbon emission system in Supplementary Fig. 1 are quantified in BBCE simulations, which are suggested by the feedback loops of Bitcoin blockchain. The whole quantitative relationships of BBCE parameters are demonstrated in Supplementary Methods.

Supplementary Methods

BBCE modelling equations

$Investment\ intensity = 40.51 \times Time \times Proportion\ of\ Chinese\ miners$ (1)

$Proportion\ of\ Chinese\ miners = IF\ THEN\ ELSE\ (Miner\ cumulative\ Profits < 0, 0.7 - 0.01 \times Time, 0.7)$ (2)

$Transaction\ fee = 0.115 \times Block\ size \times Proportion$ (3)

$Block\ size = e^{7.22+0.0215 \times Time}$ (4)

$Block\ reward = New\ block \times Mining\ reward\ halving\ mechanism$ (5)

$Bitcoin\ price = 1000 + STEP\ (6000{,}24) + STEP\ (6000{,}72) + STEP\ (12000{,}120)$ (6)

$Miner\ profit\ rate = Bitcoin\ price * (Mining\ reward\ halving\ mechanism + Transactionfee) - Total\ mining\ operating\ cost$ (7)

$Miner\ cumulative\ profits\ (t) = \int_0^t (Miner\ profit\ rate - Investment\ intensity) dt$ (8)

$GDP\ growth\ = \ Miner\ profit\ rate\ +\ Total\ mining\ operating\ cost$ (9)

$GDP(t) = \int_0^t GDP\ growth\, dt$ (10)

$Mining\ hash\ rate = 0.7 \times e^{0.0039 \times Investment\ intensity + 8.16}$ (11)

$Mining\ efficiency = e^{9.3 - 0.0018 \times Investment\ intensity \times Market\ access}$ (12)

$Mining\ power = \ Mining\ hash\ rate\ \times\ Mining\ efficiency$ (13)

$Network\ energy\ consumption\ =\ 0.7315\ \times\ Mining\ power\ \times\ Power\ usage\ effectiveness$ (14)

$Energy\ consumption\ cost\ =\ 0.05\ \times\ Network\ energy\ consumption$ (15)

$Total\ mining\ operating\ cost\ =\ Energy\ consumption\ cost\ +\ Carbon\ emission\ cost$ (16)

Coal-based $energy\ consumption$ = $Miner\ site\ selection$ × $Network\ energy\ consumption$ (17)

$Hydro\text{-}based\ energy\ consumption$ = $(1 - Miner\ site\ selection)$ × $Network\ energy\ consumption$ (18)

$Coal\text{-}based\ energy\ carbon\ emission$ = $Coal\text{-}based\ energy\ consumption$ × $Carbon\ intensity\ of\ coal\text{-}based\ energy$ (19)

$Hydro\text{-}based\ energy\ carbon\ emission$ = $Hydro\text{-}based\ energy\ consumption$ × $Carbon\ intensity\ of\ \ hydro\text{-}based\ energy$ (20)

$Total\ carbon\ emission\ (t) = \int_0^t Carbon\ emission\ flow\ dt$ (21)

$Carbon\ emission\ per\ GDP\ =\ Carbon\ emission/GDP$ (22)

$Carbontax = 0.01 \times IF\ THEN\ ELSE\ (Carbon\ emission\ perGDP > 2, 2, 1)$ (23)

$Carbon\ emission$ flow = $Coal\text{-}based\ energy\ carbon\ emission$ + $Hydro\text{-}based\ energy\ carbon\ emission$ (24)

$Carbon$ emission $cost$ = $Carbon\ tax$ ×$Carbon\ emission$ flow (25)

Supplementary Discussion

Proof-of-Work algorithm of Bitcoin blockchain. To ensure the correctness of transactions and the stability of the system, the Bitcoin blockchain technology uses the concept of Proof-of-Work (PoW)

as the current consensus algorithm. In this consensus algorithm, any new transaction that takes place in the system must be first verified and informed by a majority of miners[4]. Given that they are valid, the transactions are collected to form a block. Once a miner successfully calculates the correct hash value, the block and its corresponding hash value will be added to the blockchain, and all the local copies of the blockchain will be updated accordingly. In order to provide incentives for solving the puzzle, the consensus algorithm rewards the first miner who solved the block hash in the form of mining reward and transaction fees: on one hand, the miner receives the mining reward, which halves every 210,000 blocks, for the block it solved; on the other hand, the miner also receives the transaction fee for every successful addition of a transaction in the blockchain[5]. As a result, all the miners race to perform the PoW and calculate the correct hash value in order to collect the corresponding reward[6]. Finally, as shown in Supplementary Fig. 2, the large energy consumption of the Bitcoin blockchain has created considerable carbon emissions. It is estimated that between the period of January 1st, 2016 and June 30th, 2018, up to 13 million metric tons of CO2 emissions can be attributed to the Bitcoin blockchain.

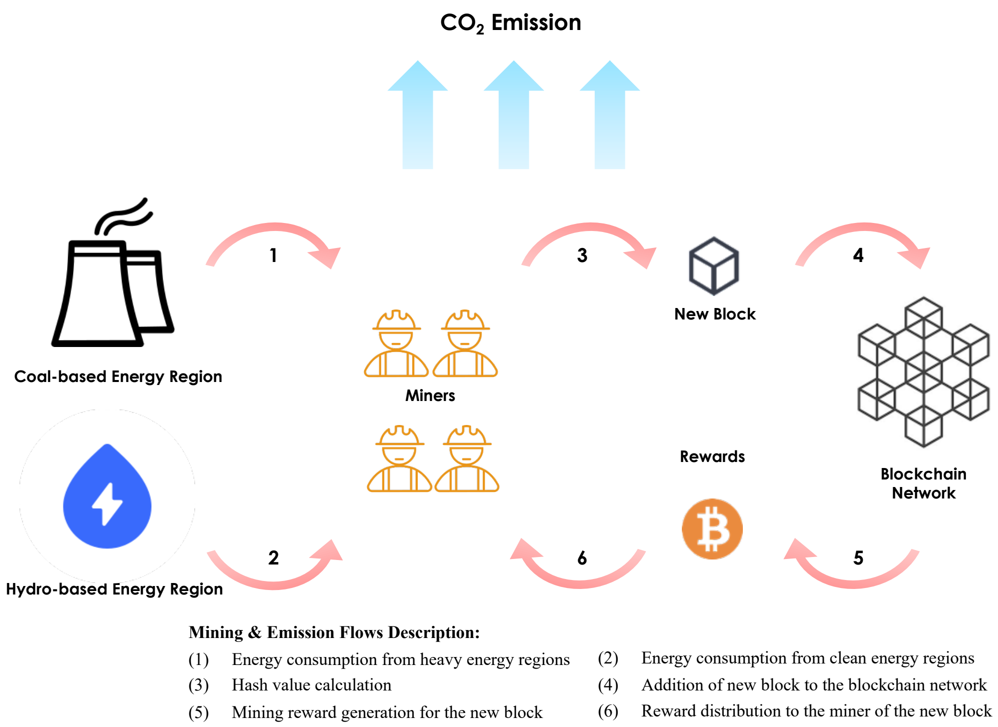

Supplementary Fig. 2 | Carbon footprint for Proof-of-Work algorithm of Bitcoin blockchain. The PoW validation process of Bitcoin blockchain involves miners solving a cryptographic puzzle to adjust the nonce

and generate a hash value lower than or equal to a certain target value, where miners earn 6.25 Bitcoin currently as new block reward. The mining and calculation process of Bitcoin blockchain requires steadily growing amount of energy due to the fierce competition between miners. Both coal-based and hydro-based energy consumed by Bitcoin miners are collected to formulate the carbon emission flows of the whole Bitcoin blockchain. The mining area distribution of Bitcoin blockchain is obtained from https://btc.com/stats. This figure is created by Yuze Li and Shangrong Jiang.

Structural suitability tests. In order to improve model transparency and conduct structural suitability tests on BBCE modelling, the System Dynamics Model Documentation and Assessment Tool (SDM) is introduced to provide documentation of models built using the Vensim modeling software. The SDM model assessment provides assessment results in three categories: model information, warnings, and potential omissions. The above information allows modelers and model readers to gain a better and specific understanding of the suitability of model in terms of its elements and confidence building tests[7].

Model Assessment Results

Model Information	Result
Total Number Of Variables	38
Total Number Of State Variables	3 (7.9%)
Total Number Of Stocks	3 (7.9%)
Total Number Of Feedback Loops No IVV (Maximum Length: 30) [3, 15]	17 (0|0|17)
Total Number Of Feedback Loops With IVV (Maximum Length: 30) [0, 0]	0 (0|0|0)
Total Number Of Causal Links	51 (0|0|51)
Total Number of Rate-to-rate Links	1
Number Of Units Used In The Model (Basic/Combined)	3/0
Total Number Of Equations Using Macros	0 (0.0%)
Variables With Source Information	0 (0.0%)
Dimensionless Unit Variables	17 (44.7%)
Function Sensitivity Parameters	0 (0.0%)
Data Lookup Tables	0 (0.0%)
Time Unit	Month
Initial Time	1
Final Time	204
Reported Time Interval	TIME STEP
Time Step	1
Model Is Fully Formulated	Yes

Warnings	Result
Variables Not In Any View	0 (0.0%)
Nonmonotonic Lookup Functions	0 (0.0%)
Cascading Lookup Functions	0 (0.0%)
Non-Zero End Sloped Lookup Functions	0 (0.0%)
Equations With If Then Else Functions	2 (5.3%)
Equations With Min Or Max Functions	0 (0.0%)
Equations With Step Pulse Or Related Functions	2 (5.3%)
Equations With Unit Errors Or Warnings	0 (0.0%)

Potential Omissions	Result
Unused Variables	0 (0.0%)
Supplementary Variables	0 (0.0%)
Supplementary Variables Being Used	0 (0.0%)
Complex Variable	1 (2.6%)
Complex Stock	0 (0.0%)

Supplementary Fig. 3 | Model assessment results of BBCE modelling. Based on the System Dynamics Model Documentation and Assessment Tool, this Figure presents the basic BBCE modelling assessment results. The whole assessment results are demonstrated in Supplementary Notes.

Supplementary Fig. 3 provides the basic BBCE modelling assessment results based on SDM tool. The structural suitability test results indicate that proposed BBCE model is able to effectively reflect the causal relationship and feedback loops in Bitcoin carbon emission system: all of the key variables are covered, and the causal relationship between variables is appropriate; the model boundary is comparatively appropriate; all the system parameters of the BBCE model have practical significance.

Reality and statistical tests. To assess the difference between real historical behaviors and BBCE modelling simulations, the reality and statistical test are performed by comparing the projected data with historical time-series data. The key Bitcoin blockchain operating time-series data from the period of January 2014 to January 2020, including actual mining hash rate and mining efficiency, are utilized to verify the parameter consistencies of BBCE modelling. We introduce R^2 to interpret the goodness of fit and parameter consistencies of BBCE modelling. Suggested by the pervious studies[8,9], the reality and statistical results are generally considered to be acceptable if the R^2 is greater than 0.9.

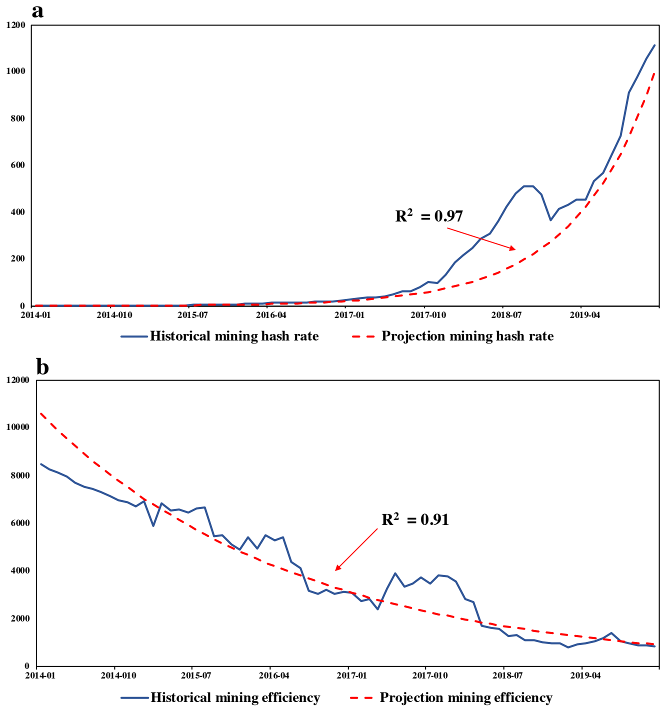

Supplementary Fig. 4 | Reality and statistical test results. This Figure illustrates the historical and projected mining hash rate (**a**) and mining efficiency (**b**) comparison results based on the actual bitcoin time-series data. We introduce R^2 to interpret the goodness of fit and parameter consistencies of BBCE modelling.

As shown in Supplementary Fig. 4, the estimated mining hash rate and mining efficiency are compared to their historical time-series data. The results show that the R^2 of estimated mining hash rate and mining efficiency are all greater than 0.9, at 0.97 and 0.91 respectively. The reality and statistical testing results indicate that the proposed BBCE model has a superior consistency between model behavior and actual situation, and also illustrate the behavioral realities of the BBCE parameters.

Sensitivity analysis. Sensitivity analysis examines the robustness of BBCE model. By adjusting the settings of important parameters, we can comment on the robustness and stability of BBCE modelling in terms of long-term trend of carbon emission flows and the carbon emission ranking of different policies. Two key constant parameters of BBCE model, i.e., power usage of efficiency (PUE) and proportions of Chinese Bitcoin servers located in coal-based region (Miner site selection) are introduced to conduct sensitivity analysis. Specifically speaking, we set PUE at 1.15 and 1.05 with respect to the utilized PUE of 1.1 in BBCE model, and Miner site selection at 43% (23% in Site Regulation scenario) and 37% (17% in Site Regulation scenario) regarding to original Miner site selection at 40% (20% in Site Regulation scenario).

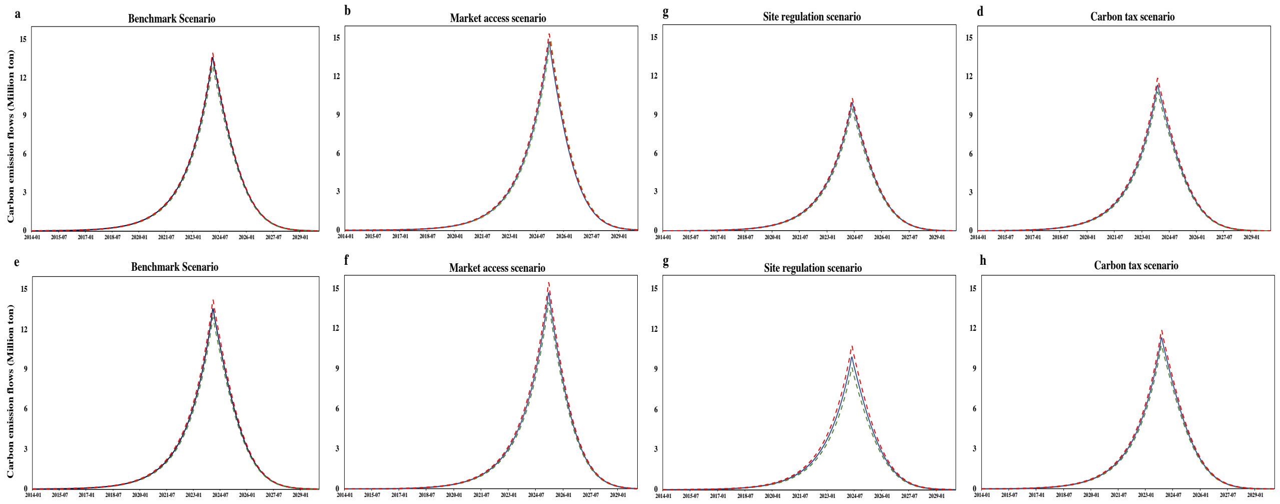

Supplementary Fig. 5 | Sensitivity analysis results. (**a**)-(**d**) provide alternative initial parameter settings of power usage efficiency (PUE) in each scenario and comparisons of the estimated carbon emission flows under different parameterizations. (**e**)-(**h**) provides alternatives initial proportions of Chinese Bitcoin servers located in coal-based region. The red dash lines in (**a**)-(**d**) denote parameterization of PUE at 1.15 and the green dash lines at 1.05. The red dash lines in (**e**)-(**h**) denote parameterization of proportions of Chinese Bitcoin servers located in coal-based region at 43% (at 23% in Site Regulation scenario) and the green dash lines at 37% (at 17% in Site Regulation scenario). The blue solid lines from (**a**)-(**d**) denote the parameterizations of PUE at 1.1 in each scenario, and that of (**e**)-(**h**) denote the parameterizations of proportions of Chinese Bitcoin servers located in coal-based region at 40% in each scenario (20% in Site regulation scenario, which are utilized in the actual BBCE modelling.

Supplementary Fig. 5 reports the sensitivity and robustness results of carbon emission flow in each scenario. It is clear that the carbon emission flow is directly proportional to the power usage efficiency (PUE) and proportions of Chinese Bitcoin servers located in coal-based region (Miner site selection). However, the long-term carbon emission trends of each sensitivity settings are consistent with that of the original BBCE parameterizations. In addition, Site Regulation scenario stable generates the lowest carbon emission flows among the 4 scenarios under different parameterizations, which indicates its stable carbon emission reduction effectiveness on the Chinese Bitcoin industry. Overall, the sensitivity test on BBCE modelling shows that a slight variation of the key parameters does not lead to remarkable changes in the model behaviors or ranking of the intended carbon reduction policies, thus indicating that the proposed BBCE model has excellent behavioral robustness and stability.

Supplementary Notes

Documentation Of BBCE modelling

Quick Links	All Variables	Variable Link Detail	Variable Types	Views	Groups	Units	Macros	Feedback Loops	Loop List No IVV	Link Polarity	View Summary	View-Variable Profile

Model Assessment Results

Model Information	Result
Total Number Of Variables	38
Total Number Of State Variables	3 (7.9%)
Total Number Of Stocks	3 (7.9%)
Total Number Of Feedback Loops No IVV (Maximum Length: 30) [3, 15]	17 (0|0|17)
Total Number Of Feedback Loops With IVV (Maximum Length: 30) [0, 0]	0 (0|0|0)
Total Number Of Causal Links	51 (0|0|51)
Total Number of Rate-to-rate Links	1
Number Of Units Used In The Model (Basic/Combined)	3/0
Total Number Of Equations Using Macros	0 (0.0%)
Variables With Source Information	0 (0.0%)
Dimensionless Unit Variables	17 (44.7%)
Function Sensitivity Parameters	0 (0.0%)
Data Lookup Tables	0 (0.0%)
Time Unit	Month
Initial Time	1
Final Time	204
Reported Time Interval	TIME STEP
Time Step	1
Model Is Fully Formulated	Yes

Warnings	Result
Variables Not In Any View	0 (0.0%)
Nonmonotonic Lookup Functions	0 (0.0%)
Cascading Lookup Functions	0 (0.0%)
Non-Zero End Sloped Lookup Functions	0 (0.0%)
Equations With If Then Else Functions	2 (5.3%)
Equations With Min Or Max Functions	0 (0.0%)
Equations With Step Pulse Or Related Functions	2 (5.3%)
Equations With Unit Errors Or Warnings	0 (0.0%)

Potential Omissions	Result
Unused Variables	0 (0.0%)
Supplementary Variables	0 (0.0%)
Supplementary Variables Being Used	0 (0.0%)
Complex Variable	1 (2.6%)
Complex Stock	0 (0.0%)

Variable Types

**L**: Level (3 / **3**)*	**SM**: Smooth (0 / **0**)*	**DE**: Delay (0 / **0**)*†	**LI**: Level Initial (0)	**I**: Initial (0 / **0**)
**C**: Constant (8 / **8**)	**F**: Flow (4 / **4**)	**A**: Auxiliary (27 / **27**)	**Sub**: Subscripts (0)	**D**: Data (0 / **0**)

G: Game (0 / 0)	T: Lookup (0 / 0)*††			

* (State Variables/Total Stocks) † Total Stocks Do Not Include Fixed Delay Variables. †† (Lookup Tables).

Views

View: View 1 (34) Variables

Groups

BBCE modelling (34)				

Quick Links:	A	B	C	D	E	F	G	H	I	J	K	L	M	N	O	P	Q	R	S	T	U	V	W	X	Y	Z

Top	(All) Variables (38 Variables)		
Group	Type	Variable Name And Description	Thumbnail
BBCE modelling	#1<br>A	**Bitcoin price (Dmnl)**<br>= 1000+STEP(6000,24)+STEP(6000,72)+STEP(12000,120)<br>**Present In 1 View:**<br>• View 1<br><br>**Used By**<br>• Miner profit rate<br><br>**Feedback Loops:** 0 (0.0%) (+) 0 [0,0] (-) 0 [0,0]	
BBCE modelling	#2<br>A	**Block hash difficulty (Dmnl)**<br>= 4320*Mining hash rate<br>**Present In 1 View:**<br>• View 1<br><br>**Used By**<br>• New block<br><br>**Feedback Loops:** 1 (5.9%) (+) 0 [0,0] (-) 0 [0,0] **(?) 1 [8,8]**	
BBCE modelling	#3<br>A	**Block reward (Dmnl)**<br>= New block*Mining reward halving mechanism<br>**Present In 1 View:**<br>• View 1<br><br>**Used By**<br>• Miner profit rate<br><br>**Feedback Loops:** 3 (17.6%) (+) 0 [0,0] (-) 0 [0,0] **(?) 3 [5,8]**	
BBCE modelling	#4<br>A	**Block size (Month)**<br>= EXP(7.22+0.0215*Time)<br>**Present In 1 View:**<br>• View 1<br><br>**Used By**<br>• Transaction fee<br><br>**Feedback Loops:** 0 (0.0%) (+) 0 [0,0] (-) 0 [0,0]	
BBCE modelling	#5<br>A	**Carbon emission cost (Month)**<br>= Carbon emission flow*Carbon tax<br>**Present In 1 View:**<br>• View 1<br><br>**Used By**<br>• Total mining operating cost	

		**Feedback Loops:** 10 (58.8%) (+) 0 [0,0] (-) 0 [0,0] **(?) 10 [6,15]**	
BBCE modelling	#6<br>F,A	**Carbon emission flow** **(Month)**<br>= "Hydro-based carbon emission"+"Coal-based carbon emission"<br>**Present In 1 View:**<br>• View 1<br><br>**Used By**<br>• Carbon emission cost<br>• Total carbon emission<br><br>**Feedback Loops:** 8 (47.1%) (+) 0 [0,0] (-) 0 [0,0] **(?) 8 [12,15]**	
BBCE modelling	#7<br>A	**Carbon emission per GDP** **(1)**<br>= Total carbon emission/GDP<br>**Present In 1 View:**<br>• View 1<br><br>**Used By**<br>• Carbon tax<br><br>**Feedback Loops:** 6 (35.3%) (+) 0 [0,0] (-) 0 [0,0] **(?) 6 [6,15]**	
BBCE modelling	#8<br>C	**Carbon intensity of coal-based energy** **(Dmnl)**<br>= 0.9<br>**Present In 1 View:**<br>• View 1<br><br>**Used By**<br>• Coal-based carbon emission<br><br>**Feedback Loops:** 0 (0.0%) (+) 0 [0,0] (-) 0 [0,0]	
BBCE modelling	#9<br>C	**Carbon intensity of hydro-based energy** **(Dmnl)**<br>= 0.2<br>**Present In 1 View:**<br>• View 1<br><br>**Used By**<br>• Hydro-based carbon emission<br><br>**Feedback Loops:** 0 (0.0%) (+) 0 [0,0] (-) 0 [0,0]	
BBCE modelling	#10<br>A	**Carbon tax** **(Dmnl)**<br>= 0.01*IF THEN ELSE(Carbon emission per GDP>2 , 2 , 1)<br>**Present In 1 View:**<br>• View 1<br><br>**Used By**<br>• Carbon emission cost<br><br>**Feedback Loops:** 6 (35.3%) (+) 0 [0,0] (-) 0 [0,0] **(?) 6 [6,15]**	
BBCE modelling	#11<br>A	**Coal-based carbon emission** **(Month)**<br>= "Carbon intensity of coal-based energy"*"Coal-based energy consumption"<br>**Present In 1 View:**<br>• View 1<br><br>**Used By**<br>• Carbon emission flow<br><br>**Feedback Loops:** 4 (23.5%) (+) 0 [0,0] (-) 0 [0,0] **(?) 4 [12,15]**	
BBCE modelling	#12<br>A	**Coal-based energy consumption** **(Month)**<br>= Miner site selection*Network energy consumption<br>**Present In 1 View:**<br>• View 1<br><br>**Used By**<br>• Coal-based carbon emission<br><br>**Feedback Loops:** 4 (23.5%) (+) 0 [0,0] (-) 0 [0,0] **(?) 4 [12,15]**	
BBCE modelling	#13<br>A	**Energy consumption cost** **(Month)**<br>= Energy price*Network energy consumption<br>**Present In 1 View:**<br>• View 1	

		**Used By**<br>• Total mining operating cost<br><br>**Feedback Loops:** 2 (11.8%) (+) 0 [0,0] (-) 0 [0,0] **(?) 2 [9,9]**	
BBCE modelling	#14<br>C	**Energy price (Dmnl)**<br>= 0.05<br>**Present In 1 View:**<br>• View 1<br><br>**Used By**<br>• Energy consumption cost<br>• Miner site selection<br><br>**Feedback Loops:** 0 (0.0%) (+) 0 [0,0] (-) 0 [0,0]	
.Control	#15<br>C	**FINAL TIME (Month)**<br>= 204<br>**Description:** The final time for the simulation.<br>**Present In 0 Views:**<br><br>**Used By**<br><br>**Feedback Loops:** 0 (0.0%) (+) 0 [0,0] (-) 0 [0,0]	
BBCE modelling	#16<br>L	**GDP (Month*Month)**<br>= $\int$ GDP growth $dt + 1.0$<br>**Present In 1 View:**<br>• View 1<br><br>**Used By**<br>• Carbon emission per GDP<br><br>**Feedback Loops:** 2 (11.8%) (+) 0 [0,0] (-) 0 [0,0] **(?) 2 [6,7]**	
BBCE modelling	#17<br>F,A	**GDP growth (Month)**<br>= Miner profit rate+Total mining operating cost<br>**Present In 1 View:**<br>• View 1<br><br>**Used By**<br>• GDP<br><br>**Feedback Loops:** 2 (11.8%) (+) 0 [0,0] (-) 0 [0,0] **(?) 2 [6,7]**	
BBCE modelling	#18<br>A	**Hydro-based carbon emission (Month)**<br>= "Carbon intensity of hydro-based energy"*"Hydro-based energy consumption"<br>**Present In 1 View:**<br>• View 1<br><br>**Used By**<br>• Carbon emission flow<br><br>**Feedback Loops:** 4 (23.5%) (+) 0 [0,0] (-) 0 [0,0] **(?) 4 [12,15]**	
BBCE modelling	#19<br>A	**Hydro-based energy consumption (Month)**<br>= Miner site selection*Network energy consumption<br>**Present In 1 View:**<br>• View 1<br><br>**Used By**<br>• Hydro-based carbon emission<br><br>**Feedback Loops:** 4 (23.5%) (+) 0 [0,0] (-) 0 [0,0] **(?) 4 [12,15]**	
.Control	#20<br>C	**INITIAL TIME (Month)**<br>= 1<br>**Description:** The initial time for the simulation.<br>**Present In 0 Views:**<br><br>**Used By**<br><br>**Feedback Loops:** 0 (0.0%) (+) 0 [0,0] (-) 0 [0,0]	
BBCE modelling	#21<br>F,A	**Investment intensity (Dmnl)**<br>= 28.36*Time*Proportion of Chinese miners/0.7<br>**Present In 1 View:**	

		• View 1<br><br>**Used By**<br>• Miner cumulative profits<br>• Mining efficiency<br>• Mining hash rate<br><br>**Feedback Loops:** 13 (76.5%) (+) 0 [0,0] (-) 0 [0,0] **(?) 13 [3,15]**	
BBCE modelling	#22<br>C	**Market assess standard for efficiency (Dmnl)**<br>= 1<br>**Present In 1 View:**<br>• View 1<br><br>**Used By**<br>• Mining efficiency<br><br>**Feedback Loops:** 0 (0.0%) (+) 0 [0,0] (-) 0 [0,0]	
BBCE modelling	#23<br>L	**Miner cumulative profits (Month*Month)**<br>= $\int$ Miner profit rate-Investment intensity dt + 0.0<br>**Present In 1 View:**<br>• View 1<br><br>**Used By**<br>• Proportion of Chinese miners<br><br>**Feedback Loops:** 15 (88.2%) (+) 0 [0,0] (-) 0 [0,0] **(?) 15 [3,15]**	
BBCE modelling	#24<br>F,A	**Miner profit rate (Month)**<br>= Bitcoin price*(Block reward+Transaction fee)-Total mining operating cost<br>**Present In 1 View:**<br>• View 1<br><br>**Used By**<br>• GDP growth<br>• Miner cumulative profits<br><br>**Feedback Loops:** 15 (88.2%) (+) 0 [0,0] (-) 0 [0,0] **(?) 15 [4,15]**	
BBCE modelling	#25<br>A	**Miner site selection (1)**<br>= 0.4+Energy price<br>**Present In 1 View:**<br>• View 1<br><br>**Used By**<br>• Coal-based energy consumption<br>• Hydro-based energy consumption<br><br>**Feedback Loops:** 0 (0.0%) (+) 0 [0,0] (-) 0 [0,0]	
BBCE modelling	#26<br>A	**Mining efficiency (Dmnl)**<br>= EXP(9.3-0.0018*Investment intensity)*Market assess standard for efficiency<br>**Present In 1 View:**<br>• View 1<br><br>**Used By**<br>• Mining power<br><br>**Feedback Loops:** 5 (29.4%) (+) 0 [0,0] (-) 0 [0,0] **(?) 5 [9,15]**	
BBCE modelling	#27<br>A	**Mining hash rate (Dmnl)**<br>= 0.7*EXP(0.0039*Investment intensity+8.16)<br>**Present In 1 View:**<br>• View 1<br><br>**Used By**<br>• Block hash difficulty<br>• Mining power<br>• New block<br><br>**Feedback Loops:** 7 (41.2%) (+) 0 [0,0] (-) 0 [0,0] **(?) 7 [7,15]**	
BBCE modelling	#28<br>A	**Mining power (1)**<br>= Mining efficiency*Mining hash rate<br>**Present In 1 View:**	

		• View 1<br><br>**Used By**<br>• Network energy consumption<br><br>**Feedback Loops:** 10 (58.8%) (+) 0 [0,0] (-) 0 [0,0] **(?) 10 [9,15]**	
BBCE modelling	#29<br>A	**Mining reward halving mechanism (Dmnl)**<br>= 25-STEP(12.5,24)-STEP(6.25,72)-STEP(3.125,120)-STEP(1.5625,168)<br>**Present In 1 View:**<br>• View 1<br><br>**Used By**<br>• Block reward<br><br>**Feedback Loops:** 0 (0.0%) (+) 0 [0,0] (-) 0 [0,0]	
BBCE modelling	#31<br>A	**Network energy consumption (Month)**<br>= Mining power*Power usage effectiveness*0.7315<br>**Present In 1 View:**<br>• View 1<br><br>**Used By**<br>• Coal-based energy consumption<br>• Energy consumption cost<br>• Hydro-based energy consumption<br><br>**Feedback Loops:** 10 (58.8%) (+) 0 [0,0] (-) 0 [0,0] **(?) 10 [9,15]**	
BBCE modelling	#32<br>A	**New block (Dmnl)**<br>= (Block hash difficulty/Mining hash rate)*Proportion of Chinese miners<br>**Present In 1 View:**<br>• View 1<br><br>**Used By**<br>• Block reward<br><br>**Feedback Loops:** 3 (17.6%) (+) 0 [0,0] (-) 0 [0,0] **(?) 3 [5,8]**	
BBCE modelling	#33<br>C	**Power usage effectiveness (Month)**<br>= 1.1<br>**Present In 1 View:**<br>• View 1<br><br>**Used By**<br>• Network energy consumption<br><br>**Feedback Loops:** 0 (0.0%) (+) 0 [0,0] (-) 0 [0,0]	
BBCE modelling	#34<br>A	**Proportion of Chinese miners (Dmnl)**<br>= IF THEN ELSE(Miner cumulative profits<0 , 0.01*(191-Time) , 0.7)<br>**Present In 1 View:**<br>• View 1<br><br>**Used By**<br>• Investment intensity<br>• New block<br>• Transaction fee<br><br>**Feedback Loops:** 15 (88.2%) (+) 0 [0,0] (-) 0 [0,0] **(?) 15 [3,15]**	
.Control	#35<br>A	**SAVEPER (Month)**<br>= TIME STEP<br>**Description:** The frequency with which output is stored.<br>**Present In 0 Views:**<br><br>**Used By**<br><br>**Feedback Loops:** 0 (0.0%) (+) 0 [0,0] (-) 0 [0,0]	
.Control	#37<br>C	**TIME STEP (Month)**<br>= 1<br>**Description:** The time step for the simulation.<br>**Present In 0 Views:**<br><br>**Used By**<br>• SAVEPER The frequency with which output is stored.	

		**Feedback Loops:** 0 (0.0%) (+) 0 [0,0] (-) 0 [0,0]	
BBCE modelling	#38<br>L	**Total carbon emission (Month*Month)**<br>= $\int$Carbon emission flow *dt* + 1.0<br>**Present In 1 View:**<br>• View 1<br><br>**Used By**<br>• Carbon emission per GDP<br><br>**Feedback Loops:** 4 (23.5%) (+) 0 [0,0] (-) 0 [0,0] **(?) 4 [15,15]**	
BBCE modelling	#39<br>A	**Total mining operating cost (Month)**<br>= Carbon emission cost+Energy consumption cost<br>**Present In 1 View:**<br>• View 1<br><br>**Used By**<br>• GDP growth<br>• Miner profit rate<br><br>**Feedback Loops:** 12 (70.6%) (+) 0 [0,0] (-) 0 [0,0] **(?) 12 [6,15]**	
BBCE modelling	#40<br>A	**Transaction fee (Month)**<br>= 0.115*Block size*Proportion of Chinese miners<br>**Present In 1 View:**<br>• View 1<br><br>**Used By**<br>• Miner profit rate<br><br>**Feedback Loops:** 1 (5.9%) (+) 0 [0,0] (-) 0 [0,0] **(?) 1 [4,4]**	

(View) View 1 (34 Variables)

Top	(View) View 1 (34 Variables)		
Group	Type	***Variable Name And Description***	Thumbnail
BBCE modelling	#1<br>A	**Bitcoin price (Dmnl)**<br>= 1000+STEP(6000,24)+STEP(6000,72)+STEP(12000,120)<br>**Present In 1 View:**<br>• View 1<br><br>**Used By**<br>• Miner profit rate<br><br>**Feedback Loops:** 0 (0.0%) (+) 0 [0,0] (-) 0 [0,0]	
BBCE modelling	#2<br>A	**Block hash difficulty (Dmnl)**<br>= 4320*Mining hash rate<br>**Present In 1 View:**<br>• View 1<br><br>**Used By**<br>• New block<br><br>**Feedback Loops:** 1 (5.9%) (+) 0 [0,0] (-) 0 [0,0] **(?) 1 [8,8]**	
BBCE modelling	#3<br>A	**Block reward (Dmnl)**<br>= New block*Mining reward halving mechanism<br>**Present In 1 View:**<br>• View 1<br><br>**Used By**<br>• Miner profit rate<br><br>**Feedback Loops:** 3 (17.6%) (+) 0 [0,0] (-) 0 [0,0] **(?) 3 [5,8]**	
BBCE modelling	#4<br>A	**Block size (Month)**<br>= EXP(7.22+0.0215*Time)<br>**Present In 1 View:**<br>• View 1<br><br>**Used By**<br>• Transaction fee	

		Feedback Loops: 0 (0.0%) (+) 0 [0,0] (-) 0 [0,0]	
BBCE modelling	#5<br>A	**Carbon emission cost** **(Month)**<br>= Carbon emission flow*Carbon tax<br>**Present In 1 View:**<br>• View 1<br><br>**Used By**<br>• Total mining operating cost<br><br>**Feedback Loops:** 10 (58.8%) (+) 0 [0,0] (-) 0 [0,0] **(?) 10 [6,15]**	
BBCE modelling	#6<br>F,A	**Carbon emission flow** **(Month)**<br>= "Hydro-based carbon emission"+"Coal-based carbon emission"<br>**Present In 1 View:**<br>• View 1<br><br>**Used By**<br>• Carbon emission cost<br>• Total carbon emission<br><br>**Feedback Loops:** 8 (47.1%) (+) 0 [0,0] (-) 0 [0,0] **(?) 8 [12,15]**	
BBCE modelling	#7<br>A	**Carbon emission per GDP** **(1)**<br>= Total carbon emission/GDP<br>**Present In 1 View:**<br>• View 1<br><br>**Used By**<br>• Carbon tax<br><br>**Feedback Loops:** 6 (35.3%) (+) 0 [0,0] (-) 0 [0,0] **(?) 6 [6,15]**	
BBCE modelling	#8<br>C	**Carbon intensity of coal-based energy** **(Dmnl)**<br>= 0.9<br>**Present In 1 View:**<br>• View 1<br><br>**Used By**<br>• Coal-based carbon emission<br><br>**Feedback Loops:** 0 (0.0%) (+) 0 [0,0] (-) 0 [0,0]	
BBCE modelling	#9<br>C	**Carbon intensity of hydro-based energy** **(Dmnl)**<br>= 0.2<br>**Present In 1 View:**<br>• View 1<br><br>**Used By**<br>• Hydro-based carbon emission<br><br>**Feedback Loops:** 0 (0.0%) (+) 0 [0,0] (-) 0 [0,0]	
BBCE modelling	#10<br>A	**Carbon tax** **(Dmnl)**<br>= 0.01*IF THEN ELSE(Carbon emission per GDP>2 , 2 , 1)<br>**Present In 1 View:**<br>• View 1<br><br>**Used By**<br>• Carbon emission cost<br><br>**Feedback Loops:** 6 (35.3%) (+) 0 [0,0] (-) 0 [0,0] **(?) 6 [6,15]**	
BBCE modelling	#11<br>A	**Coal-based carbon emission** **(Month)**<br>= "Carbon intensity of coal-based energy"*"Coal-based energy consumption"<br>**Present In 1 View:**<br>• View 1<br><br>**Used By**<br>• Carbon emission flow<br><br>**Feedback Loops:** 4 (23.5%) (+) 0 [0,0] (-) 0 [0,0] **(?) 4 [12,15]**	
BBCE modelling	#12<br>A	**Coal-based energy consumption** **(Month)**<br>= Miner site selection*Network energy consumption<br>**Present In 1 View:**<br>• View 1	

		**Used By**<br>• Coal-based carbon emission<br><br>**Feedback Loops:** 4 (23.5%) (+) 0 [0,0] (-) 0 [0,0] **(?) 4 [12,15]**	
BBCE modelling	#13<br>A	**Energy consumption cost** **(Month)**<br>= Energy price*Network energy consumption<br>**Present In 1 View:**<br>• View 1<br><br>**Used By**<br>• Total mining operating cost<br><br>**Feedback Loops:** 2 (11.8%) (+) 0 [0,0] (-) 0 [0,0] **(?) 2 [9,9]**	
BBCE modelling	#14<br>C	**Energy price** **(Dmnl)**<br>= 0.05<br>**Present In 1 View:**<br>• View 1<br><br>**Used By**<br>• Energy consumption cost<br>• Miner site selection<br><br>**Feedback Loops:** 0 (0.0%) (+) 0 [0,0] (-) 0 [0,0]	
BBCE modelling	#16<br>L	**GDP** **(Month*Month)**<br>$= \int$GDP growth $dt + 1.0$<br>**Present In 1 View:**<br>• View 1<br><br>**Used By**<br>• Carbon emission per GDP<br><br>**Feedback Loops:** 2 (11.8%) (+) 0 [0,0] (-) 0 [0,0] **(?) 2 [6,7]**	
BBCE modelling	#17<br>F,A	**GDP growth** **(Month)**<br>= Miner profit rate+Total mining operating cost<br>**Present In 1 View:**<br>• View 1<br><br>**Used By**<br>• GDP<br><br>**Feedback Loops:** 2 (11.8%) (+) 0 [0,0] (-) 0 [0,0] **(?) 2 [6,7]**	
BBCE modelling	#18<br>A	**Hydro-based carbon emission** **(Month)**<br>= "Carbon intensity of hydro-based energy"*"Hydro-based energy consumption"<br>**Present In 1 View:**<br>• View 1<br><br>**Used By**<br>• Carbon emission flow<br><br>**Feedback Loops:** 4 (23.5%) (+) 0 [0,0] (-) 0 [0,0] **(?) 4 [12,15]**	
BBCE modelling	#19<br>A	**Hydro-based energy consumption** **(Month)**<br>= Miner site selection*Network energy consumption<br>**Present In 1 View:**<br>• View 1<br><br>**Used By**<br>• Hydro-based carbon emission<br><br>**Feedback Loops:** 4 (23.5%) (+) 0 [0,0] (-) 0 [0,0] **(?) 4 [12,15]**	
BBCE modelling	#21<br>F,A	**Investment intensity** **(Dmnl)**<br>= 28.36*Time*Proportion of Chinese miners/0.7<br>**Present In 1 View:**<br>• View 1<br><br>**Used By**<br>• Miner cumulative profits<br>• Mining efficiency<br>• Mining hash rate	

		**Feedback Loops:** 13 (76.5%) (+) 0 [0,0] (-) 0 [0,0] **(?) 13 [3,15]**	
BBCE modelling	#22<br>C	**Market assess standard for efficiency (Dmnl)**<br>= 1<br>**Present In 1 View:**<br>• View 1<br><br>**Used By**<br>• Mining efficiency<br><br>**Feedback Loops:** 0 (0.0%) (+) 0 [0,0] (-) 0 [0,0]	
BBCE modelling	#23<br>L	**Miner cumulative profits (Month*Month)**<br>= $\int$Miner profit rate-Investment intensity dt + 0.0<br>**Present In 1 View:**<br>• View 1<br><br>**Used By**<br>• Proportion of Chinese miners<br><br>**Feedback Loops:** 15 (88.2%) (+) 0 [0,0] (-) 0 [0,0] **(?) 15 [3,15]**	
BBCE modelling	#24<br>F,A	**Miner profit rate (Month)**<br>= Bitcoin price*(Block reward+Transaction fee)-Total mining operating cost<br>**Present In 1 View:**<br>• View 1<br><br>**Used By**<br>• GDP growth<br>• Miner cumulative profits<br><br>**Feedback Loops:** 15 (88.2%) (+) 0 [0,0] (-) 0 [0,0] **(?) 15 [4,15]**	
BBCE modelling	#25<br>A	**Miner site selection (1)**<br>= 0.4+Energy price<br>**Present In 1 View:**<br>• View 1<br><br>**Used By**<br>• Coal-based energy consumption<br>• Hydro-based energy consumption<br><br>**Feedback Loops:** 0 (0.0%) (+) 0 [0,0] (-) 0 [0,0]	
BBCE modelling	#26<br>A	**Mining efficiency (Dmnl)**<br>= EXP(9.3-0.0018*Investment intensity)*Market assess standard for efficiency<br>**Present In 1 View:**<br>• View 1<br><br>**Used By**<br>• Mining power<br><br>**Feedback Loops:** 5 (29.4%) (+) 0 [0,0] (-) 0 [0,0] **(?) 5 [9,15]**	
BBCE modelling	#27<br>A	**Mining hash rate (Dmnl)**<br>= 0.7*EXP(0.0039*Investment intensity+8.16)<br>**Present In 1 View:**<br>• View 1<br><br>**Used By**<br>• Block hash difficulty<br>• Mining power<br>• New block<br><br>**Feedback Loops:** 7 (41.2%) (+) 0 [0,0] (-) 0 [0,0] **(?) 7 [7,15]**	
BBCE modelling	#28<br>A	**Mining power (1)**<br>= Mining efficiency*Mining hash rate<br>**Present In 1 View:**<br>• View 1<br><br>**Used By**<br>• Network energy consumption	

		**Feedback Loops:** 10 (58.8%) (+) 0 [0,0] (-) 0 [0,0] **(?) 10 [9,15]**	
BBCE modelling	#29<br>A	**Mining reward halving mechanism (Dmnl)**<br>= 25-STEP(12.5,24)-STEP(6.25,72)-STEP(3.125,120)-STEP(1.5625,168)<br>**Present In 1 View:**<br>• View 1<br><br>**Used By**<br>• Block reward<br><br>**Feedback Loops:** 0 (0.0%) (+) 0 [0,0] (-) 0 [0,0]	
BBCE modelling	#31<br>A	**Network energy consumption (Month)**<br>= Mining power*Power usage effectiveness*0.7315<br>**Present In 1 View:**<br>• View 1<br><br>**Used By**<br>• Coal-based energy consumption<br>• Energy consumption cost<br>• Hydro-based energy consumption<br><br>**Feedback Loops:** 10 (58.8%) (+) 0 [0,0] (-) 0 [0,0] **(?) 10 [9,15]**	
BBCE modelling	#32<br>A	**New block (Dmnl)**<br>= (Block hash difficulty/Mining hash rate)*Proportion of Chinese miners<br>**Present In 1 View:**<br>• View 1<br><br>**Used By**<br>• Block reward<br><br>**Feedback Loops:** 3 (17.6%) (+) 0 [0,0] (-) 0 [0,0] **(?) 3 [5,8]**	
BBCE modelling	#33<br>C	**Power usage effectiveness (Month)**<br>= 1.1<br>**Present In 1 View:**<br>• View 1<br><br>**Used By**<br>• Network energy consumption<br><br>**Feedback Loops:** 0 (0.0%) (+) 0 [0,0] (-) 0 [0,0]	
BBCE modelling	#34<br>A	**Proportion of Chinese miners (Dmnl)**<br>= IF THEN ELSE(Miner cumulative profits<0 , 0.01*(191-Time) , 0.7)<br>**Present In 1 View:**<br>• View 1<br><br>**Used By**<br>• Investment intensity<br>• New block<br>• Transaction fee<br><br>**Feedback Loops:** 15 (88.2%) (+) 0 [0,0] (-) 0 [0,0] **(?) 15 [3,15]**	
BBCE modelling	#38<br>L	**Total carbon emission (Month*Month)**<br>= $\int$ Carbon emission flow dt + 1.0<br>**Present In 1 View:**<br>• View 1<br><br>**Used By**<br>• Carbon emission per GDP<br><br>**Feedback Loops:** 4 (23.5%) (+) 0 [0,0] (-) 0 [0,0] **(?) 4 [15,15]**	
BBCE modelling	#39<br>A	**Total mining operating cost (Month)**<br>= Carbon emission cost+Energy consumption cost<br>**Present In 1 View:**<br>• View 1<br><br>**Used By**<br>• GDP growth<br>• Miner profit rate<br><br>**Feedback Loops:** 12 (70.6%) (+) 0 [0,0] (-) 0 [0,0] **(?) 12 [6,15]**	

BBCE modelling	#40 A	**Transaction fee** **(Month)**<br>= 0.115*Block size*Proportion of Chinese miners<br>**Present In 1 View:**<br>• View 1<br><br>**Used By**<br>• Miner profit rate<br><br>**Feedback Loops:** 1 (5.9%) (+) 0 [0,0] (-) 0 [0,0] **(?) 1 [4,4]**	

Top	(Group) BBCE modelling (34 Variables)		
Group	Type	*Variable Name And Description*	Thumbnail
BBCE modelling	#1 A	**Bitcoin price** **(Dmnl)**<br>= 1000+STEP(6000,24)+STEP(6000,72)+STEP(12000,120)<br>**Present In 1 View:**<br>• View 1<br><br>**Used By**<br>• Miner profit rate<br><br>**Feedback Loops:** 0 (0.0%) (+) 0 [0,0] (-) 0 [0,0]	
BBCE modelling	#2 A	**Block hash difficulty** **(Dmnl)**<br>= 4320*Mining hash rate<br>**Present In 1 View:**<br>• View 1<br><br>**Used By**<br>• New block<br><br>**Feedback Loops:** 1 (5.9%) (+) 0 [0,0] (-) 0 [0,0] **(?) 1 [8,8]**	
BBCE modelling	#3 A	**Block reward** **(Dmnl)**<br>= New block*Mining reward halving mechanism<br>**Present In 1 View:**<br>• View 1<br><br>**Used By**<br>• Miner profit rate<br><br>**Feedback Loops:** 3 (17.6%) (+) 0 [0,0] (-) 0 [0,0] **(?) 3 [5,8]**	
BBCE modelling	#4 A	**Block size** **(Month)**<br>= EXP(7.22+0.0215*Time)<br>**Present In 1 View:**<br>• View 1<br><br>**Used By**<br>• Transaction fee<br><br>**Feedback Loops:** 0 (0.0%) (+) 0 [0,0] (-) 0 [0,0]	
BBCE modelling	#5 A	**Carbon emission cost** **(Month)**<br>= Carbon emission flow*Carbon tax<br>**Present In 1 View:**<br>• View 1<br><br>**Used By**<br>• Total mining operating cost<br><br>**Feedback Loops:** 10 (58.8%) (+) 0 [0,0] (-) 0 [0,0] **(?) 10 [6,15]**	
BBCE modelling	#6 F,A	**Carbon emission flow** **(Month)**<br>= "Hydro-based carbon emission"+"Coal-based carbon emission"<br>**Present In 1 View:**<br>• View 1<br><br>**Used By**<br>• Carbon emission cost<br>• Total carbon emission<br><br>**Feedback Loops:** 8 (47.1%) (+) 0 [0,0] (-) 0 [0,0] **(?) 8 [12,15]**	
BBCE modelling	#7 A	**Carbon emission per GDP** **(1)**<br>= Total carbon emission/GDP	

		**Present In 1 View:**<br>• View 1<br><br>**Used By**<br>• Carbon tax<br><br>**Feedback Loops:** 6 (35.3%) (+) 0 [0,0] (-) 0 [0,0] **(?) 6 [6,15]**	
BBCE modelling	#8<br>C	**Carbon intensity of coal-based energy (Dmnl)**<br>= 0.9<br>**Present In 1 View:**<br>• View 1<br><br>**Used By**<br>• Coal-based carbon emission<br><br>**Feedback Loops:** 0 (0.0%) (+) 0 [0,0] (-) 0 [0,0]	
BBCE modelling	#9<br>C	**Carbon intensity of hydro-based energy (Dmnl)**<br>= 0.2<br>**Present In 1 View:**<br>• View 1<br><br>**Used By**<br>• Hydro-based carbon emission<br><br>**Feedback Loops:** 0 (0.0%) (+) 0 [0,0] (-) 0 [0,0]	
BBCE modelling	#10<br>A	**Carbon tax (Dmnl)**<br>= 0.01*IF THEN ELSE(Carbon emission per GDP>2 , 2 , 1)<br>**Present In 1 View:**<br>• View 1<br><br>**Used By**<br>• Carbon emission cost<br><br>**Feedback Loops:** 6 (35.3%) (+) 0 [0,0] (-) 0 [0,0] **(?) 6 [6,15]**	
BBCE modelling	#11<br>A	**Coal-based carbon emission (Month)**<br>= "Carbon intensity of coal-based energy"*"Coal-based energy consumption"<br>**Present In 1 View:**<br>• View 1<br><br>**Used By**<br>• Carbon emission flow<br><br>**Feedback Loops:** 4 (23.5%) (+) 0 [0,0] (-) 0 [0,0] **(?) 4 [12,15]**	
BBCE modelling	#12<br>A	**Coal-based energy consumption (Month)**<br>= Miner site selection*Network energy consumption<br>**Present In 1 View:**<br>• View 1<br><br>**Used By**<br>• Coal-based carbon emission<br><br>**Feedback Loops:** 4 (23.5%) (+) 0 [0,0] (-) 0 [0,0] **(?) 4 [12,15]**	
BBCE modelling	#13<br>A	**Energy consumption cost (Month)**<br>= Energy price*Network energy consumption<br>**Present In 1 View:**<br>• View 1<br><br>**Used By**<br>• Total mining operating cost<br><br>**Feedback Loops:** 2 (11.8%) (+) 0 [0,0] (-) 0 [0,0] **(?) 2 [9,9]**	
BBCE modelling	#14<br>C	**Energy price (Dmnl)**<br>= 0.05<br>**Present In 1 View:**<br>• View 1<br><br>**Used By**<br>• Energy consumption cost<br>• Miner site selection	

		**Feedback Loops:** 0 (0.0%) (+) 0 [0,0] (-) 0 [0,0]	
BBCE modelling	#16 L	**GDP (Month*Month)**<br>= $\int$GDP growth dt + 1.0<br>**Present In 1 View:**<br>• View 1<br><br>**Used By**<br>• Carbon emission per GDP<br><br>**Feedback Loops:** 2 (11.8%) (+) 0 [0,0] (-) 0 [0,0] **(?) 2 [6,7]**	
BBCE modelling	#17 F,A	**GDP growth (Month)**<br>= Miner profit rate+Total mining operating cost<br>**Present In 1 View:**<br>• View 1<br><br>**Used By**<br>• GDP<br><br>**Feedback Loops:** 2 (11.8%) (+) 0 [0,0] (-) 0 [0,0] **(?) 2 [6,7]**	
BBCE modelling	#18 A	**Hydro-based carbon emission (Month)**<br>= "Carbon intensity of hydro-based energy"*"Hydro-based energy consumption"<br>**Present In 1 View:**<br>• View 1<br><br>**Used By**<br>• Carbon emission flow<br><br>**Feedback Loops:** 4 (23.5%) (+) 0 [0,0] (-) 0 [0,0] **(?) 4 [12,15]**	
BBCE modelling	#19 A	**Hydro-based energy consumption (Month)**<br>= Miner site selection*Network energy consumption<br>**Present In 1 View:**<br>• View 1<br><br>**Used By**<br>• Hydro-based carbon emission<br><br>**Feedback Loops:** 4 (23.5%) (+) 0 [0,0] (-) 0 [0,0] **(?) 4 [12,15]**	
BBCE modelling	#21 F,A	**Investment intensity (Dmnl)**<br>= 28.36*Time*Proportion of Chinese miners/0.7<br>**Present In 1 View:**<br>• View 1<br><br>**Used By**<br>• Miner cumulative profits<br>• Mining efficiency<br>• Mining hash rate<br><br>**Feedback Loops:** 13 (76.5%) (+) 0 [0,0] (-) 0 [0,0] **(?) 13 [3,15]**	
BBCE modelling	#22 C	**Market assess standard for efficiency (Dmnl)**<br>= 1<br>**Present In 1 View:**<br>• View 1<br><br>**Used By**<br>• Mining efficiency<br><br>**Feedback Loops:** 0 (0.0%) (+) 0 [0,0] (-) 0 [0,0]	
BBCE modelling	#23 L	**Miner cumulative profits (Month*Month)**<br>= $\int$Miner profit rate-Investment intensity dt + 0.0<br>**Present In 1 View:**<br>• View 1<br><br>**Used By**<br>• Proportion of Chinese miners<br><br>**Feedback Loops:** 15 (88.2%) (+) 0 [0,0] (-) 0 [0,0] **(?) 15 [3,15]**	
BBCE modelling	#24	**Miner profit rate (Month)**	

	F,A	= Bitcoin price*(Block reward+Transaction fee)-Total mining operating cost<br>**Present In 1 View:**<br>• View 1<br><br>**Used By**<br>• GDP growth<br>• Miner cumulative profits<br><br>**Feedback Loops:** 15 (88.2%) (+) 0 [0,0] (-) 0 [0,0] **(?) 15 [4,15]**	
BBCE modelling	#25 A	**Miner site selection (1)**<br>= 0.4+Energy price<br>**Present In 1 View:**<br>• View 1<br><br>**Used By**<br>• Coal-based energy consumption<br>• Hydro-based energy consumption<br><br>**Feedback Loops:** 0 (0.0%) (+) 0 [0,0] (-) 0 [0,0]	
BBCE modelling	#26 A	**Mining efficiency (Dmnl)**<br>= EXP(9.3-0.0018*Investment intensity)*Market assess standard for efficiency<br>**Present In 1 View:**<br>• View 1<br><br>**Used By**<br>• Mining power<br><br>**Feedback Loops:** 5 (29.4%) (+) 0 [0,0] (-) 0 [0,0] **(?) 5 [9,15]**	
BBCE modelling	#27 A	**Mining hash rate (Dmnl)**<br>= 0.7*EXP(0.0039*Investment intensity+8.16)<br>**Present In 1 View:**<br>• View 1<br><br>**Used By**<br>• Block hash difficulty<br>• Mining power<br>• New block<br><br>**Feedback Loops:** 7 (41.2%) (+) 0 [0,0] (-) 0 [0,0] **(?) 7 [7,15]**	
BBCE modelling	#28 A	**Mining power (1)**<br>= Mining efficiency*Mining hash rate<br>**Present In 1 View:**<br>• View 1<br><br>**Used By**<br>• Network energy consumption<br><br>**Feedback Loops:** 10 (58.8%) (+) 0 [0,0] (-) 0 [0,0] **(?) 10 [9,15]**	
BBCE modelling	#29 A	**Mining reward halving mechanism (Dmnl)**<br>= 25-STEP(12.5,24)-STEP(6.25,72)-STEP(3.125,120)-STEP(1.5625,168)<br>**Present In 1 View:**<br>• View 1<br><br>**Used By**<br>• Block reward<br><br>**Feedback Loops:** 0 (0.0%) (+) 0 [0,0] (-) 0 [0,0]	
BBCE modelling	#31 A	**Network energy consumption (Month)**<br>= Mining power*Power usage effectiveness*0.7315<br>**Present In 1 View:**<br>• View 1<br><br>**Used By**<br>• Coal-based energy consumption<br>• Energy consumption cost<br>• Hydro-based energy consumption<br><br>**Feedback Loops:** 10 (58.8%) (+) 0 [0,0] (-) 0 [0,0] **(?) 10 [9,15]**	
BBCE modelling	#32	**New block (Dmnl)**	

	A	= (Block hash difficulty/Mining hash rate)*Proportion of Chinese miners<br>**Present In 1 View:**<br>• View 1<br><br>**Used By**<br>• Block reward<br><br>**Feedback Loops:** 3 (17.6%) (+) 0 [0,0] (-) 0 [0,0] **(?) 3 [5,8]**	
BBCE modelling	#33<br>C	**Power usage effectiveness (Month)**<br>= 1.1<br>**Present In 1 View:**<br>• View 1<br><br>**Used By**<br>• Network energy consumption<br><br>**Feedback Loops:** 0 (0.0%) (+) 0 [0,0] (-) 0 [0,0]	
BBCE modelling	#34<br>A	**Proportion of Chinese miners (Dmnl)**<br>= IF THEN ELSE(Miner cumulative profits<0 , 0.01*(191-Time) , 0.7)<br>**Present In 1 View:**<br>• View 1<br><br>**Used By**<br>• Investment intensity<br>• New block<br>• Transaction fee<br><br>**Feedback Loops:** 15 (88.2%) (+) 0 [0,0] (-) 0 [0,0] **(?) 15 [3,15]**	
BBCE modelling	#38<br>L	**Total carbon emission (Month*Month)**<br>= $\int$ Carbon emission flow $dt + 1.0$<br>**Present In 1 View:**<br>• View 1<br><br>**Used By**<br>• Carbon emission per GDP<br><br>**Feedback Loops:** 4 (23.5%) (+) 0 [0,0] (-) 0 [0,0] **(?) 4 [15,15]**	
BBCE modelling	#39<br>A	**Total mining operating cost (Month)**<br>= Carbon emission cost+Energy consumption cost<br>**Present In 1 View:**<br>• View 1<br><br>**Used By**<br>• GDP growth<br>• Miner profit rate<br><br>**Feedback Loops:** 12 (70.6%) (+) 0 [0,0] (-) 0 [0,0] **(?) 12 [6,15]**	
BBCE modelling	#40<br>A	**Transaction fee (Month)**<br>= 0.115*Block size*Proportion of Chinese miners<br>**Present In 1 View:**<br>• View 1<br><br>**Used By**<br>• Miner profit rate<br><br>**Feedback Loops:** 1 (5.9%) (+) 0 [0,0] (-) 0 [0,0] **(?) 1 [4,4]**	

Top	**(Type) Level (3 Variables)**		
Group	Type	***Variable Name And Description***	Thumbnail
BBCE modelling	#16<br>L	**GDP (Month*Month)**<br>= $\int$ GDP growth $dt + 1.0$<br>**Present In 1 View:**<br>• View 1<br><br>**Used By**<br>• Carbon emission per GDP<br><br>**Feedback Loops:** 2 (11.8%) (+) 0 [0,0] (-) 0 [0,0] **(?) 2 [6,7]**	

BBCE modelling	#23<br>L	**Miner cumulative profits** **(Month*Month)**<br>$= \int$ Miner profit rate-Investment intensity dt + 0.0<br>**Present In 1 View:**<br>• View 1<br><br>**Used By**<br>• Proportion of Chinese miners<br><br>**Feedback Loops:** 15 (88.2%) (+) 0 [0,0] (-) 0 [0,0] **(?) 15 [3,15]**	
BBCE modelling	#38<br>L	**Total carbon emission** **(Month*Month)**<br>$= \int$ Carbon emission flow dt + 1.0<br>**Present In 1 View:**<br>• View 1<br><br>**Used By**<br>• Carbon emission per GDP<br><br>**Feedback Loops:** 4 (23.5%) (+) 0 [0,0] (-) 0 [0,0] **(?) 4 [15,15]**	

Top	(Type) Smooth (0 Variables)		
Group	Type	***Variable Name And Description***	Thumbnail

Top	(Type) Delay (0 Variables)		
Group	Type	***Variable Name And Description***	Thumbnail

Top	(Type) Level Initial (0 Variables)		
Group	Type	***Variable Name And Description***	Thumbnail

Top	(Type) Initial (0 Variables)		
Group	Type	***Variable Name And Description***	Thumbnail

Top	(Type) Constant (5 Variables)		
Group	Type	***Variable Name And Description***	Thumbnail
BBCE modelling	#8<br>C	**Carbon intensity of coal-based energy** **(Dmnl)**<br>= 0.9<br>**Present In 1 View:**<br>• View 1<br><br>**Used By**<br>• Coal-based carbon emission<br><br>**Feedback Loops:** 0 (0.0%) (+) 0 [0,0] (-) 0 [0,0]	
BBCE modelling	#9<br>C	**Carbon intensity of hydro-based energy** **(Dmnl)**<br>= 0.2<br>**Present In 1 View:**<br>• View 1<br><br>**Used By**<br>• Hydro-based carbon emission<br><br>**Feedback Loops:** 0 (0.0%) (+) 0 [0,0] (-) 0 [0,0]	
BBCE modelling	#14<br>C	**Energy price** **(Dmnl)**<br>= 0.05<br>**Present In 1 View:**<br>• View 1<br><br>**Used By**<br>• Energy consumption cost<br>• Miner site selection<br><br>**Feedback Loops:** 0 (0.0%) (+) 0 [0,0] (-) 0 [0,0]	
BBCE modelling	#22<br>C	**Market assess standard for efficiency** **(Dmnl)**<br>= 1<br>**Present In 1 View:**<br>• View 1<br><br>**Used By**<br>• Mining efficiency	

		**Feedback Loops:** 0 (0.0%) (+) 0 [0,0] (-) 0 [0,0]	
BBCE modelling	#33<br>C	**Power usage effectiveness (Month)**<br>= 1.1<br>**Present In 1 View:**<br>• View 1<br><br>**Used By**<br>• Network energy consumption<br><br>**Feedback Loops:** 0 (0.0%) (+) 0 [0,0] (-) 0 [0,0]	

Top	(Type) Flow (4 Variables)		
Group	Type	***Variable Name And Description***	Thumbnail
BBCE modelling	#6<br>F,A	**Carbon emission flow (Month)**<br>= "Hydro-based carbon emission"+"Coal-based carbon emission"<br>**Present In 1 View:**<br>• View 1<br><br>**Used By**<br>• Carbon emission cost<br>• Total carbon emission<br><br>**Feedback Loops:** 8 (47.1%) (+) 0 [0,0] (-) 0 [0,0] **(?) 8 [12,15]**	
BBCE modelling	#17<br>F,A	**GDP growth (Month)**<br>= Miner profit rate+Total mining operating cost<br>**Present In 1 View:**<br>• View 1<br><br>**Used By**<br>• GDP<br><br>**Feedback Loops:** 2 (11.8%) (+) 0 [0,0] (-) 0 [0,0] **(?) 2 [6,7]**	
BBCE modelling	#21<br>F,A	**Investment intensity (Dmnl)**<br>= 28.36*Time*Proportion of Chinese miners/0.7<br>**Present In 1 View:**<br>• View 1<br><br>**Used By**<br>• Miner cumulative profits<br>• Mining efficiency<br>• Mining hash rate<br><br>**Feedback Loops:** 13 (76.5%) (+) 0 [0,0] (-) 0 [0,0] **(?) 13 [3,15]**	
BBCE modelling	#24<br>F,A	**Miner profit rate (Month)**<br>= Bitcoin price*(Block reward+Transaction fee)-Total mining operating cost<br>**Present In 1 View:**<br>• View 1<br><br>**Used By**<br>• GDP growth<br>• Miner cumulative profits<br><br>**Feedback Loops:** 15 (88.2%) (+) 0 [0,0] (-) 0 [0,0] **(?) 15 [4,15]**	

Top	(Type) Auxiliary (26 Variables)		
Group	Type	***Variable Name And Description***	Thumbnail
BBCE modelling	#1<br>A	**Bitcoin price (Dmnl)**<br>= 1000+STEP(6000,24)+STEP(6000,72)+STEP(12000,120)<br>**Present In 1 View:**<br>• View 1<br><br>**Used By**<br>• Miner profit rate<br><br>**Feedback Loops:** 0 (0.0%) (+) 0 [0,0] (-) 0 [0,0]	
BBCE modelling	#2<br>A	**Block hash difficulty (Dmnl)**<br>= 4320*Mining hash rate<br>**Present In 1 View:**<br>• View 1	

		**Used By**<br>• New block<br><br>**Feedback Loops:** 1 (5.9%) (+) 0 [0,0] (-) 0 [0,0] **(?) 1 [8,8]**	
BBCE modelling	#3<br>A	**Block reward (Dmnl)**<br>= New block*Mining reward halving mechanism<br>**Present In 1 View:**<br>• View 1<br><br>**Used By**<br>• Miner profit rate<br><br>**Feedback Loops:** 3 (17.6%) (+) 0 [0,0] (-) 0 [0,0] **(?) 3 [5,8]**	
BBCE modelling	#4<br>A	**Block size (Month)**<br>= EXP(7.22+0.0215*Time)<br>**Present In 1 View:**<br>• View 1<br><br>**Used By**<br>• Transaction fee<br><br>**Feedback Loops:** 0 (0.0%) (+) 0 [0,0] (-) 0 [0,0]	
BBCE modelling	#5<br>A	**Carbon emission cost (Month)**<br>= Carbon emission flow*Carbon tax<br>**Present In 1 View:**<br>• View 1<br><br>**Used By**<br>• Total mining operating cost<br><br>**Feedback Loops:** 10 (58.8%) (+) 0 [0,0] (-) 0 [0,0] **(?) 10 [6,15]**	
BBCE modelling	#6<br>F,A	**Carbon emission flow (Month)**<br>= "Hydro-based carbon emission"+"Coal-based carbon emission"<br>**Present In 1 View:**<br>• View 1<br><br>**Used By**<br>• Carbon emission cost<br>• Total carbon emission<br><br>**Feedback Loops:** 8 (47.1%) (+) 0 [0,0] (-) 0 [0,0] **(?) 8 [12,15]**	
BBCE modelling	#7<br>A	**Carbon emission per GDP (1)**<br>= Total carbon emission/GDP<br>**Present In 1 View:**<br>• View 1<br><br>**Used By**<br>• Carbon tax<br><br>**Feedback Loops:** 6 (35.3%) (+) 0 [0,0] (-) 0 [0,0] **(?) 6 [6,15]**	
BBCE modelling	#10<br>A	**Carbon tax (Dmnl)**<br>= 0.01*IF THEN ELSE(Carbon emission per GDP>2 , 2 , 1)<br>**Present In 1 View:**<br>• View 1<br><br>**Used By**<br>• Carbon emission cost<br><br>**Feedback Loops:** 6 (35.3%) (+) 0 [0,0] (-) 0 [0,0] **(?) 6 [6,15]**	
BBCE modelling	#11<br>A	**Coal-based carbon emission (Month)**<br>= "Carbon intensity of coal-based energy"*"Coal-based energy consumption"<br>**Present In 1 View:**<br>• View 1<br><br>**Used By**<br>• Carbon emission flow<br><br>**Feedback Loops:** 4 (23.5%) (+) 0 [0,0] (-) 0 [0,0] **(?) 4 [12,15]**	
BBCE modelling	#12	**Coal-based energy consumption (Month)**	

	A	= Miner site selection*Network energy consumption<br>**Present In 1 View:**<br>• View 1<br><br>**Used By**<br>• Coal-based carbon emission<br><br>**Feedback Loops:** 4 (23.5%) (+) 0 [0,0] (-) 0 [0,0] **(?) 4 [12,15]**	
BBCE modelling	#13<br>A	**Energy consumption cost (Month)**<br>= Energy price*Network energy consumption<br>**Present In 1 View:**<br>• View 1<br><br>**Used By**<br>• Total mining operating cost<br><br>**Feedback Loops:** 2 (11.8%) (+) 0 [0,0] (-) 0 [0,0] **(?) 2 [9,9]**	
BBCE modelling	#17<br>F,A	**GDP growth (Month)**<br>= Miner profit rate+Total mining operating cost<br>**Present In 1 View:**<br>• View 1<br><br>**Used By**<br>• GDP<br><br>**Feedback Loops:** 2 (11.8%) (+) 0 [0,0] (-) 0 [0,0] **(?) 2 [6,7]**	
BBCE modelling	#18<br>A	**Hydro-based carbon emission (Month)**<br>= "Carbon intensity of hydro-based energy"*"Hydro-based energy consumption"<br>**Present In 1 View:**<br>• View 1<br><br>**Used By**<br>• Carbon emission flow<br><br>**Feedback Loops:** 4 (23.5%) (+) 0 [0,0] (-) 0 [0,0] **(?) 4 [12,15]**	
BBCE modelling	#19<br>A	**Hydro-based energy consumption (Month)**<br>= Miner site selection*Network energy consumption<br>**Present In 1 View:**<br>• View 1<br><br>**Used By**<br>• Hydro-based carbon emission<br><br>**Feedback Loops:** 4 (23.5%) (+) 0 [0,0] (-) 0 [0,0] **(?) 4 [12,15]**	
BBCE modelling	#21<br>F,A	**Investment intensity (Dmnl)**<br>= 28.36*Time*Proportion of Chinese miners/0.7<br>**Present In 1 View:**<br>• View 1<br><br>**Used By**<br>• Miner cumulative profits<br>• Mining efficiency<br>• Mining hash rate<br><br>**Feedback Loops:** 13 (76.5%) (+) 0 [0,0] (-) 0 [0,0] **(?) 13 [3,15]**	
BBCE modelling	#24<br>F,A	**Miner profit rate (Month)**<br>= Bitcoin price*(Block reward+Transaction fee)-Total mining operating cost<br>**Present In 1 View:**<br>• View 1<br><br>**Used By**<br>• GDP growth<br>• Miner cumulative profits<br><br>**Feedback Loops:** 15 (88.2%) (+) 0 [0,0] (-) 0 [0,0] **(?) 15 [4,15]**	
BBCE modelling	#25<br>A	**Miner site selection (1)**<br>= 0.4+Energy price<br>**Present In 1 View:**<br>• View 1	

		**Used By**<br>• Coal-based energy consumption<br>• Hydro-based energy consumption<br><br>**Feedback Loops:** 0 (0.0%) (+) 0 [0,0] (-) 0 [0,0]	
BBCE modelling	#26<br>A	**Mining efficiency (Dmnl)**<br>= EXP(9.3-0.0018*Investment intensity)*Market assess standard for efficiency<br>**Present In 1 View:**<br>• View 1<br><br>**Used By**<br>• Mining power<br><br>**Feedback Loops:** 5 (29.4%) (+) 0 [0,0] (-) 0 [0,0] **(?) 5 [9,15]**	
BBCE modelling	#27<br>A	**Mining hash rate (Dmnl)**<br>= 0.7*EXP(0.0039*Investment intensity+8.16)<br>**Present In 1 View:**<br>• View 1<br><br>**Used By**<br>• Block hash difficulty<br>• Mining power<br>• New block<br><br>**Feedback Loops:** 7 (41.2%) (+) 0 [0,0] (-) 0 [0,0] **(?) 7 [7,15]**	
BBCE modelling	#28<br>A	**Mining power (1)**<br>= Mining efficiency*Mining hash rate<br>**Present In 1 View:**<br>• View 1<br><br>**Used By**<br>• Network energy consumption<br><br>**Feedback Loops:** 10 (58.8%) (+) 0 [0,0] (-) 0 [0,0] **(?) 10 [9,15]**	
BBCE modelling	#29<br>A	**Mining reward halving mechanism (Dmnl)**<br>= 25-STEP(12.5,24)-STEP(6.25,72)-STEP(3.125,120)-STEP(1.5625,168)<br>**Present In 1 View:**<br>• View 1<br><br>**Used By**<br>• Block reward<br><br>**Feedback Loops:** 0 (0.0%) (+) 0 [0,0] (-) 0 [0,0]	
BBCE modelling	#31<br>A	**Network energy consumption (Month)**<br>= Mining power*Power usage effectiveness*0.7315<br>**Present In 1 View:**<br>• View 1<br><br>**Used By**<br>• Coal-based energy consumption<br>• Energy consumption cost<br>• Hydro-based energy consumption<br><br>**Feedback Loops:** 10 (58.8%) (+) 0 [0,0] (-) 0 [0,0] **(?) 10 [9,15]**	
BBCE modelling	#32<br>A	**New block (Dmnl)**<br>= (Block hash difficulty/Mining hash rate)*Proportion of Chinese miners<br>**Present In 1 View:**<br>• View 1<br><br>**Used By**<br>• Block reward<br><br>**Feedback Loops:** 3 (17.6%) (+) 0 [0,0] (-) 0 [0,0] **(?) 3 [5,8]**	
BBCE modelling	#34<br>A	**Proportion of Chinese miners (Dmnl)**<br>= IF THEN ELSE(Miner cumulative profits<0 , 0.01*(191-Time) , 0.7)<br>**Present In 1 View:**<br>• View 1<br><br>**Used By**	

		• Investment intensity<br>• New block<br>• Transaction fee<br><br>**Feedback Loops:** 15 (88.2%) (+) 0 [0,0] (-) 0 [0,0] **(?) 15 [3,15]**	
BBCE modelling	#39 A	**Total mining operating cost (Month)**<br>= Carbon emission cost+Energy consumption cost<br>**Present In 1 View:**<br>• View 1<br><br>**Used By**<br>• GDP growth<br>• Miner profit rate<br><br>**Feedback Loops:** 12 (70.6%) (+) 0 [0,0] (-) 0 [0,0] **(?) 12 [6,15]**	
BBCE modelling	#40 A	**Transaction fee (Month)**<br>= 0.115*Block size*Proportion of Chinese miners<br>**Present In 1 View:**<br>• View 1<br><br>**Used By**<br>• Miner profit rate<br><br>**Feedback Loops:** 1 (5.9%) (+) 0 [0,0] (-) 0 [0,0] **(?) 1 [4,4]**	

Top	(Type) Subscripts (0 Variables)		
Group	Type	***Variable Name And Description***	Thumbnail

Top	(Type) Data (0 Variables)		
Group	Type	***Variable Name And Description***	Thumbnail

Top	(Type) Game (0 Variables)		
Group	Type	***Variable Name And Description***	Thumbnail

Top	(Type) Lookup (0 Variables)	
Group	Type	***Variable Name And Description***

Top

**Quick Links:**	A	B	C	D	E	F	G	H	I	J	K	L	M	N	O	P	Q	R	S	T	U	V	W	X	Y	Z

All Variables (38)

Group	Type	**Variable**
BBCE modelling	A	Bitcoin price (Dmnl)
BBCE modelling	A	Block hash difficulty (Dmnl)
BBCE modelling	A	Block reward (Dmnl)
BBCE modelling	A	Block size (Month)
BBCE modelling	A	Carbon emission cost (Month)
BBCE modelling	F,A	Carbon emission flow (Month)
BBCE modelling	A	Carbon emission per GDP (1)
BBCE modelling	C	Carbon intensity of coal-based energy (Dmnl)
BBCE modelling	C	Carbon intensity of hydro-based energy (Dmnl)
BBCE modelling	A	Carbon tax (Dmnl)
BBCE modelling	A	Coal-based carbon emission (Month)
BBCE modelling	A	Coal-based energy consumption (Month)
BBCE modelling	A	Energy consumption cost (Month)
BBCE modelling	C	Energy price (Dmnl)
.Control	C	FINAL TIME (Month)
BBCE modelling	L	GDP (Month*Month)
BBCE modelling	F,A	GDP growth (Month)
BBCE modelling	A	Hydro-based carbon emission (Month)
BBCE modelling	A	Hydro-based energy consumption (Month)

.Control	C	INITIAL TIME (Month)
BBCE modelling	F,A	Investment intensity (Dmnl)
BBCE modelling	C	Market assess standard for efficiency (Dmnl)
BBCE modelling	L	Miner cumulative profits (Month*Month)
BBCE modelling	F,A	Miner profit rate (Month)
BBCE modelling	A	Miner site selection (1)
BBCE modelling	A	Mining efficiency (Dmnl)
BBCE modelling	A	Mining hash rate (Dmnl)
BBCE modelling	A	Mining power (1)
BBCE modelling	A	Mining reward halving mechanism (Dmnl)
BBCE modelling	A	Network energy consumption (Month)
BBCE modelling	A	New block (Dmnl)
BBCE modelling	C	Power usage effectiveness (Month)
BBCE modelling	A	Proportion of Chinese miners (Dmnl)
.Control	A	SAVEPER (Month)
.Control	C	TIME STEP (Month)
BBCE modelling	L	Total carbon emission (Month*Month)
BBCE modelling	A	Total mining operating cost (Month)
BBCE modelling	A	Transaction fee (Month)

Top

Variable Link Detail (38)

Group	Type	Variable	In/Out Counts	In/Out Ratio	In Links by Polarity	Out Links by Polarity
BBCE modelling	F,A	Miner profit rate (Month)	4 | 2	2.00	0| 0| 4	0| 0| 2
BBCE modelling	A	Proportion of Chinese miners (Dmnl)	2 | 3	0.67	0| 0| 2	0| 0| 3
BBCE modelling	A	Network energy consumption (Month)	2 | 3	0.67	0| 0| 2	0| 0| 3
BBCE modelling	F,A	Investment intensity (Dmnl)	2 | 3	0.67	0| 0| 2	0| 0| 3
BBCE modelling	A	Total mining operating cost (Month)	2 | 2	1.00	0| 0| 2	0| 0| 2
BBCE modelling	A	New block (Dmnl)	3 | 1	3.00	0| 0| 3	0| 0| 1
BBCE modelling	A	Mining hash rate (Dmnl)	1 | 3	0.33	0| 0| 1	0| 0| 3
BBCE modelling	F,A	Carbon emission flow (Month)	2 | 2	1.00	0| 0| 2	0| 0| 2
BBCE modelling	A	Transaction fee (Month)	2 | 1	2.00	0| 0| 2	0| 0| 1
BBCE modelling	A	Mining power (1)	2 | 1	2.00	0| 0| 2	0| 0| 1
BBCE modelling	A	Mining efficiency (Dmnl)	2 | 1	2.00	0| 0| 2	0| 0| 1
BBCE modelling	A	Miner site selection (1)	1 | 2	0.50	0| 0| 1	0| 0| 2
BBCE modelling	L	Miner cumulative profits (Month*Month)	2 | 1	2.00	0| 0| 2	0| 0| 1
BBCE modelling	A	Hydro-based energy consumption (Month)	2 | 1	2.00	0| 0| 2	0| 0| 1
BBCE modelling	A	Hydro-based carbon emission (Month)	2 | 1	2.00	0| 0| 2	0| 0| 1
BBCE modelling	F,A	GDP growth (Month)	2 | 1	2.00	0| 0| 2	0| 0| 1
BBCE modelling	A	Energy consumption cost (Month)	2 | 1	2.00	0| 0| 2	0| 0| 1
BBCE modelling	A	Coal-based energy consumption (Month)	2 | 1	2.00	0| 0| 2	0| 0| 1

BBCE modelling	A	Coal-based carbon emission (Month)	2 | 1	2.00	0| 0| 2	0| 0| 1
BBCE modelling	A	Carbon emission per GDP (1)	2 | 1	2.00	0| 0| 2	0| 0| 1
BBCE modelling	A	Carbon emission cost (Month)	2 | 1	2.00	0| 0| 2	0| 0| 1
BBCE modelling	A	Block reward (Dmnl)	2 | 1	2.00	0| 0| 2	0| 0| 1
BBCE modelling	L	Total carbon emission (Month*Month)	1 | 1	1.00	0| 0| 1	0| 0| 1
BBCE modelling	L	GDP (Month*Month)	1 | 1	1.00	0| 0| 1	0| 0| 1
BBCE modelling	C	Energy price (Dmnl)	0 | 2	0.00	0| 0| 0	0| 0| 2
BBCE modelling	A	Carbon tax (Dmnl)	1 | 1	1.00	0| 0| 1	0| 0| 1
BBCE modelling	A	Block size (Month)	1 | 1	1.00	0| 0| 1	0| 0| 1
BBCE modelling	A	Block hash difficulty (Dmnl)	1 | 1	1.00	0| 0| 1	0| 0| 1
.Control	C	TIME STEP (Month)	0 | 1	0.00	0| 0| 0	0| 0| 1
.Control	A	SAVEPER (Month)	1 | 0	∞	0| 0| 1	0| 0| 0
BBCE modelling	C	Power usage effectiveness (Month)	0 | 1	0.00	0| 0| 0	0| 0| 1
BBCE modelling	A	Mining reward halving mechanism (Dmnl)	0 | 1	0.00	0| 0| 0	0| 0| 1
BBCE modelling	C	Market assess standard for efficiency (Dmnl)	0 | 1	0.00	0| 0| 0	0| 0| 1
BBCE modelling	C	Carbon intensity of hydro-based energy (Dmnl)	0 | 1	0.00	0| 0| 0	0| 0| 1
BBCE modelling	C	Carbon intensity of coal-based energy (Dmnl)	0 | 1	0.00	0| 0| 0	0| 0| 1
BBCE modelling	A	Bitcoin price (Dmnl)	0 | 1	0.00	0| 0| 0	0| 0| 1
.Control	C	INITIAL TIME (Month)	(0| 0)	∞	0| 0| 0	0| 0| 0
.Control	C	FINAL TIME (Month)	(0| 0)	∞	0| 0| 0	0| 0| 0

Top

Supplementary Variables (0)

Group	Type	**Variable**

Top

Supplementary Variables Being Used (0)

Group	Type	**Variable**

Top

Unused Variables (0)

Group	Type	**Variable**

Top

Nonmonotonic Lookup Functions (0)

Group	Type	**Variable**

Top

Non-Zero End Sloped Lookup Functions (0)

Group	Type	**Variable**	**Non-Zero**

Top

Cascading Lookup Functions (0)

Group	Type	**Variable**

Top

**Quick Links:**	A	B	C	D	E	F	G	H	I	J	K	L	M	N	O	P	Q	R	S	T	U	V	W	X	Y	Z

Equations With Step Pulse Or Related Functions (2)

Group	Type	**Variable**
BBCE modelling	A	Bitcoin price (Dmnl)
BBCE modelling	A	Mining reward halving mechanism (Dmnl)

Top

**Quick Links:**	A	B	C	D	E	F	G	H	I	J	K	L	M	N	O	P	Q	R	S	T	U	V	W	X	Y	Z

Equations With If Then Else Functions (2)

Group	Type	**Variable**
BBCE modelling	A	Carbon tax (Dmnl)
BBCE modelling	A	Proportion of Chinese miners (Dmnl)

Top

Equations With Min Or Max Functions (0)

Group	Type	Variable

Top

Complex Variable (Richardson's Rule Threshold = 3) (1)

Group	Type	Variable	Complexity
BBCE modelling	F,A	Miner profit rate (Month)	4

Top

Complex Stock (0)

Group	Type	Variable

Top

Variables With Source Information (0)

Group	Type	Variable

Top

Quick Links:	A	B	C	D	E	F	G	H	I	J	K	L	M	N	O	P	Q	R	S	T	U	V	W	X	Y	Z

Variables With Dimensionless Units (17)

Group	Type	Variable
BBCE modelling	A	Bitcoin price (Dmnl)
BBCE modelling	A	Block hash difficulty (Dmnl)
BBCE modelling	A	Block reward (Dmnl)
BBCE modelling	A	Carbon emission per GDP (1)
BBCE modelling	C	Carbon intensity of coal-based energy (Dmnl)
BBCE modelling	C	Carbon intensity of hydro-based energy (Dmnl)
BBCE modelling	A	Carbon tax (Dmnl)
BBCE modelling	C	Energy price (Dmnl)
BBCE modelling	F,A	Investment intensity (Dmnl)
BBCE modelling	C	Market assess standard for efficiency (Dmnl)
BBCE modelling	A	Miner site selection (1)
BBCE modelling	A	Mining efficiency (Dmnl)
BBCE modelling	A	Mining hash rate (Dmnl)
BBCE modelling	A	Mining power (1)
BBCE modelling	A	Mining reward halving mechanism (Dmnl)
BBCE modelling	A	New block (Dmnl)
BBCE modelling	A	Proportion of Chinese miners (Dmnl)

Top

Function Sensitivity Parameters (0)

Group	Type	Variable

Top

Data Lookup Tables (0)

Group	Type	Variable

Top

Variables Not In Any View (0)

Group	Type	Variable

Top

Equations With Unit Errors Or Warnings (0)

Group	Type	Variable

Top

Units (3/0)

Units	Type	Alternates
Dmnl	Basic	[1]
Month	Basic	
Month*Month	Basic	

Top

Feedback Loops (17|0 Maximum Length: 30 [3,15] | [0,0])

Group	Type	Variable	Loops	+	-	+/- Ratio	?	Loops (IVV)	+	-	+/- Ratio	?
BBCE modelling	L	Miner cumulative profits (Month*Month)	15 (88.2%)	0 [0,0]	0 [0,0]	NA	**15** [3,15]	0 (0.0%)	0 [0,0]	0 [0,0]	NA	**0** [0,0]
BBCE modelling	F,A	Miner profit rate (Month)	15 (88.2%)	0 [0,0]	0 [0,0]	NA	**15** [4,15]	0 (0.0%)	0 [0,0]	0 [0,0]	NA	**0** [0,0]
BBCE modelling	A	Proportion of Chinese miners (Dmnl)	15 (88.2%)	0 [0,0]	0 [0,0]	NA	**15** [3,15]	0 (0.0%)	0 [0,0]	0 [0,0]	NA	**0** [0,0]
BBCE	F,A	Investment	13 (76.5%)	0 [0,0]	0 [0,0]	NA	**13** [3,15]	0 (0.0%)	0 [0,0]	0 [0,0]	NA	**0** [0,0]

modelling		intensity (Dmnl)										
BBCE modelling	A	Total mining operating cost (Month)	12 (70.6%)	0 [0,0]	0 [0,0]	NA	**12** [6,15]	0 (0.0%)	0 [0,0]	0 [0,0]	NA	**0** [0,0]
BBCE modelling	A	Carbon emission cost (Month)	10 (58.8%)	0 [0,0]	0 [0,0]	NA	**10** [6,15]	0 (0.0%)	0 [0,0]	0 [0,0]	NA	**0** [0,0]
BBCE modelling	A	Mining power (1)	10 (58.8%)	0 [0,0]	0 [0,0]	NA	**10** [9,15]	0 (0.0%)	0 [0,0]	0 [0,0]	NA	**0** [0,0]
BBCE modelling	A	Network energy consumption (Month)	10 (58.8%)	0 [0,0]	0 [0,0]	NA	**10** [9,15]	0 (0.0%)	0 [0,0]	0 [0,0]	NA	**0** [0,0]
BBCE modelling	F,A	Carbon emission flow (Month)	8 (47.1%)	0 [0,0]	0 [0,0]	NA	**8** [12,15]	0 (0.0%)	0 [0,0]	0 [0,0]	NA	**0** [0,0]
BBCE modelling	A	Mining hash rate (Dmnl)	7 (41.2%)	0 [0,0]	0 [0,0]	NA	**7** [7,15]	0 (0.0%)	0 [0,0]	0 [0,0]	NA	**0** [0,0]
BBCE modelling	A	Carbon emission per GDP (1)	6 (35.3%)	0 [0,0]	0 [0,0]	NA	**6** [6,15]	0 (0.0%)	0 [0,0]	0 [0,0]	NA	**0** [0,0]
BBCE modelling	A	Carbon tax (Dmnl)	6 (35.3%)	0 [0,0]	0 [0,0]	NA	**6** [6,15]	0 (0.0%)	0 [0,0]	0 [0,0]	NA	**0** [0,0]
BBCE modelling	A	Mining efficiency (Dmnl)	5 (29.4%)	0 [0,0]	0 [0,0]	NA	**5** [9,15]	0 (0.0%)	0 [0,0]	0 [0,0]	NA	**0** [0,0]
BBCE modelling	A	Coal-based carbon emission (Month)	4 (23.5%)	0 [0,0]	0 [0,0]	NA	**4** [12,15]	0 (0.0%)	0 [0,0]	0 [0,0]	NA	**0** [0,0]
BBCE modelling	A	Coal-based energy consumption (Month)	4 (23.5%)	0 [0,0]	0 [0,0]	NA	**4** [12,15]	0 (0.0%)	0 [0,0]	0 [0,0]	NA	**0** [0,0]
BBCE modelling	A	Hydro-based carbon emission (Month)	4 (23.5%)	0 [0,0]	0 [0,0]	NA	**4** [12,15]	0 (0.0%)	0 [0,0]	0 [0,0]	NA	**0** [0,0]
BBCE modelling	A	Hydro-based energy consumption (Month)	4 (23.5%)	0 [0,0]	0 [0,0]	NA	**4** [12,15]	0 (0.0%)	0 [0,0]	0 [0,0]	NA	**0** [0,0]
BBCE modelling	L	Total carbon emission (Month*Month)	4 (23.5%)	0 [0,0]	0 [0,0]	NA	**4** [15,15]	0 (0.0%)	0 [0,0]	0 [0,0]	NA	**0** [0,0]
BBCE modelling	A	Block reward (Dmnl)	3 (17.6%)	0 [0,0]	0 [0,0]	NA	**3** [5,8]	0 (0.0%)	0 [0,0]	0 [0,0]	NA	**0** [0,0]
BBCE modelling	A	New block (Dmnl)	3 (17.6%)	0 [0,0]	0 [0,0]	NA	**3** [5,8]	0 (0.0%)	0 [0,0]	0 [0,0]	NA	**0** [0,0]
BBCE modelling	A	Energy consumption cost (Month)	2 (11.8%)	0 [0,0]	0 [0,0]	NA	**2** [9,9]	0 (0.0%)	0 [0,0]	0 [0,0]	NA	**0** [0,0]
BBCE modelling	L	GDP (Month*Month)	2 (11.8%)	0 [0,0]	0 [0,0]	NA	**2** [6,7]	0 (0.0%)	0 [0,0]	0 [0,0]	NA	**0** [0,0]
BBCE modelling	F,A	GDP growth (Month)	2 (11.8%)	0 [0,0]	0 [0,0]	NA	**2** [6,7]	0 (0.0%)	0 [0,0]	0 [0,0]	NA	**0** [0,0]
BBCE modelling	A	Block hash difficulty (Dmnl)	1 (5.9%)	0 [0,0]	0 [0,0]	NA	**1** [8,8]	0 (0.0%)	0 [0,0]	0 [0,0]	NA	**0** [0,0]
BBCE modelling	A	Transaction fee (Month)	1 (5.9%)	0 [0,0]	0 [0,0]	NA	**1** [4,4]	0 (0.0%)	0 [0,0]	0 [0,0]	NA	**0** [0,0]
BBCE modelling	A	Bitcoin price (Dmnl)	0 (0.0%)	0 [0,0]	0 [0,0]	NA	**0** [0,0]	0 (0.0%)	0 [0,0]	0 [0,0]	NA	**0** [0,0]
BBCE modelling	A	Block size (Month)	0 (0.0%)	0 [0,0]	0 [0,0]	NA	**0** [0,0]	0 (0.0%)	0 [0,0]	0 [0,0]	NA	**0** [0,0]

BBCE modelling	C	Carbon intensity of coal-based energy (Dmnl)	0 (0.0%)	0 [0,0]	0 [0,0]	NA	**0** [0,0]	0 (0.0%)	0 [0,0]	0 [0,0]	NA	**0** [0,0]
BBCE modelling	C	Carbon intensity of hydro-based energy (Dmnl)	0 (0.0%)	0 [0,0]	0 [0,0]	NA	**0** [0,0]	0 (0.0%)	0 [0,0]	0 [0,0]	NA	**0** [0,0]
BBCE modelling	C	Energy price (Dmnl)	0 (0.0%)	0 [0,0]	0 [0,0]	NA	**0** [0,0]	0 (0.0%)	0 [0,0]	0 [0,0]	NA	**0** [0,0]
.Control	C	FINAL TIME (Month)	0 (0.0%)	0 [0,0]	0 [0,0]	NA	**0** [0,0]	0 (0.0%)	0 [0,0]	0 [0,0]	NA	**0** [0,0]
.Control	C	INITIAL TIME (Month)	0 (0.0%)	0 [0,0]	0 [0,0]	NA	**0** [0,0]	0 (0.0%)	0 [0,0]	0 [0,0]	NA	**0** [0,0]
BBCE modelling	C	Market assess standard for efficiency (Dmnl)	0 (0.0%)	0 [0,0]	0 [0,0]	NA	**0** [0,0]	0 (0.0%)	0 [0,0]	0 [0,0]	NA	**0** [0,0]
BBCE modelling	A	Miner site selection (1)	0 (0.0%)	0 [0,0]	0 [0,0]	NA	**0** [0,0]	0 (0.0%)	0 [0,0]	0 [0,0]	NA	**0** [0,0]
BBCE modelling	A	Mining reward halving mechanism (Dmnl)	0 (0.0%)	0 [0,0]	0 [0,0]	NA	**0** [0,0]	0 (0.0%)	0 [0,0]	0 [0,0]	NA	**0** [0,0]
BBCE modelling	C	Power usage effectiveness (Month)	0 (0.0%)	0 [0,0]	0 [0,0]	NA	**0** [0,0]	0 (0.0%)	0 [0,0]	0 [0,0]	NA	**0** [0,0]
.Control	A	SAVEPER (Month)	0 (0.0%)	0 [0,0]	0 [0,0]	NA	**0** [0,0]	0 (0.0%)	0 [0,0]	0 [0,0]	NA	**0** [0,0]
.Control	C	TIME STEP (Month)	0 (0.0%)	0 [0,0]	0 [0,0]	NA	**0** [0,0]	0 (0.0%)	0 [0,0]	0 [0,0]	NA	**0** [0,0]

Top

Macros (0)

Name	Macro Definition	Expanded Macro Definition

Top

Positive Polarity Causal Links (0)

Cause	Effect	**Polarity**

Top

Negative Polarity Causal Links (0)

Cause	Effect	**Polarity**

Top

**Quick**	A	B	C	D	E	F	G	H	I	J	K	L	M	N	O	P	Q	R	S	T	U	V	W	X	Y	Z

Links:																										

Function-based Polarity Causal Links (51)

Cause	Effect	Polarity
Bitcoin price	Miner profit rate	?
Block hash difficulty	New block	?
Block reward	Miner profit rate	?
Block size	Transaction fee	?
Carbon emission cost	Total mining operating cost	?
Carbon emission flow	Carbon emission cost	?
Carbon emission flow	Total carbon emission	?
Carbon emission per GDP	Carbon tax	If Then Else Switch
Carbon intensity of coal-based energy	Coal-based carbon emission	?
Carbon intensity of hydro-based energy	Hydro-based carbon emission	?
Carbon tax	Carbon emission cost	?
Coal-based carbon emission	Carbon emission flow	?
Coal-based energy consumption	Coal-based carbon emission	?
Energy consumption cost	Total mining operating cost	?
Energy price	Energy consumption cost	?
Energy price	Miner site selection	?
GDP	Carbon emission per GDP	?
GDP growth	GDP	?
Hydro-based carbon emission	Carbon emission flow	?
Hydro-based energy consumption	Hydro-based carbon emission	?
Investment intensity	Miner cumulative profits	?
Investment intensity	Mining efficiency	?
Investment intensity	Mining hash rate	?
Market assess standard for efficiency	Mining efficiency	?
Miner cumulative profits	Proportion of Chinese miners	If Then Else Switch
Miner profit rate	GDP growth	?
Miner profit rate	Miner cumulative profits	?
Miner site selection	Coal-based energy consumption	?
Miner site selection	Hydro-based energy consumption	?
Mining efficiency	Mining power	?
Mining hash rate	Block hash difficulty	?
Mining hash rate	Mining power	?
Mining hash rate	New block	?
Mining power	Network energy consumption	?
Mining reward halving mechanism	Block reward	?
Network energy consumption	Coal-based energy consumption	?
Network energy consumption	Energy consumption cost	?
Network energy consumption	Hydro-based energy consumption	?
New block	Block reward	?
Power usage effectiveness	Network energy consumption	?
Proportion of Chinese miners	Investment intensity	?
Proportion of Chinese miners	New block	?
Proportion of Chinese miners	Transaction fee	?
Time	Block size	?
Time	Investment intensity	?
Time	Proportion of Chinese miners	?
TIME STEP	SAVEPER	?
Total carbon emission	Carbon emission per GDP	?
Total mining operating cost	GDP growth	?
Total mining operating cost	Miner profit rate	?
Transaction fee	Miner profit rate	?

Top

Rate-to-rate Links (1)

Cause	Effect
Miner profit rate	GDP growth

Top

View-Variable Profile

View	View-Variable Profile
View 1	34 vars (85%)

Top

List Of 1 views and their 34 Variables

	View 1	
**Total:**	**34**	**Total:**
New block (In 1 View)		New block (In 1 View)
Coal-based energy consumption (In 1 View)		Coal-based energy consumption (In 1 View)
Carbon emission flow (In 1 View)		Carbon emission flow (In 1 View)
Block hash difficulty (In 1 View)		Block hash difficulty (In 1 View)
Block reward (In 1 View)		Block reward (In 1 View)
Mining efficiency (In 1 View)		Mining efficiency (In 1 View)
Total carbon emission (In 1 View)		Total carbon emission (In 1 View)
Carbon tax (In 1 View)		Carbon tax (In 1 View)
Hydro-based carbon emission (In 1 View)		Hydro-based carbon emission (In 1 View)
Power usage effectiveness (In 1 View)		Power usage effectiveness (In 1 View)
Energy consumption cost (In 1 View)		Energy consumption cost (In 1 View)
Hydro-based energy consumption (In 1 View)		Hydro-based energy consumption (In 1 View)
GDP (In 1 View)		GDP (In 1 View)
Carbon emission per GDP (In 1 View)		Carbon emission per GDP (In 1 View)
Market assess standard for efficiency (In 1 View)		Market assess standard for efficiency (In 1 View)
GDP growth (In 1 View)		GDP growth (In 1 View)
Block size (In 1 View)		Block size (In 1 View)
Miner cumulative profits (In 1 View)		Miner cumulative profits (In 1 View)
Investment intensity (In 1 View)		Investment intensity (In 1 View)
Proportion of Chinese miners (In 1 View)		Proportion of Chinese miners (In 1 View)
Network energy consumption (In 1 View)		Network energy consumption (In 1 View)
Mining reward halving mechanism (In 1 View)		Mining reward halving mechanism (In 1 View)
Transaction fee (In 1 View)		Transaction fee (In 1 View)
Carbon emission cost (In 1 View)		Carbon emission cost (In 1 View)
Coal-based carbon emission (In 1 View)		Coal-based carbon emission (In 1 View)
Mining hash rate (In 1 View)		Mining hash rate (In 1 View)
Carbon intensity of hydro-based energy (In 1 View)		Carbon intensity of hydro-based energy (In 1 View)
Mining power (In 1 View)		Mining power (In 1 View)
Energy price (In 1 View)		Energy price (In 1 View)
Miner site selection (In 1 View)		Miner site selection (In 1 View)
Total mining operating cost (In 1 View)		Total mining operating cost (In 1 View)
Miner profit rate (In 1 View)		Miner profit rate (In 1 View)
Bitcoin price (In 1 View)		Bitcoin price (In 1 View)
Carbon intensity of coal-based energy (In 1 View)		Carbon intensity of coal-based energy (In 1 View)
**Total:**	**34**	**Total:**
	View 1	

Supplementary References

1. Küfeoğlu, S., & Özkuran, M. Bitcoin mining: A global review of energy and power demand. Energy Res. Soc. Sci. 58, 101273 (2019).
2. Stoll, C., Klaaßen, L., & Gallersdörfer, U. The carbon footprint of bitcoin. Joule 3, 1647-1661 (2019).
3. Cheng, Z., Li, L., & Liu, J. Industrial structure, technical progress and carbon intensity in China's provinces. Renew. Sust. Energ. Rev. 81, 2935-2946 (2018).
4. Houy, N. Rational mining limits Bitcoin emissions. Nat. Clim. Chang. 9, 655 (2019).
5. Conti, M., Kumar, E. S., Lal, C., & Ruj, S. A survey on security and privacy issues of bitcoin. IEEE Commun. Surv. Tutor. 20, 3416-3452 (2018).
6. Tschorsch, F., & Scheuermann, B. Bitcoin and beyond: A technical survey on decentralized digital currencies. IEEE Commun. Surv. Tutor. 18, 2084-2123 (2016).
7. Martinez-Moyano, I. J. Documentation for model transparency. Syst. Dyn. Rev. 28(2), 199-208 (2012).
8. Oliva, R. Model calibration as a testing strategy for system dynamics models. Eur. J. Oper. Res. 151(3), 552-568 (2003)
9. Summers, H. D., Rees, P., Holton, M. D., Brown, M. R., Chappell, S. C., Smith, P. J., & Errington, R. J. Statistical analysis of nanoparticle dosing in a dynamic cellular system. Nat. Nanotechnol. 6(3), 170-174 (2011).
