# Supplementary material for: Policy assessments for the carbon emission flows and sustainability of Bitcoin blockchain operation in China
Source: Nat Commun. 2021 Apr 6;12:1938. doi: 10.1038/s41467-021-22256-3 (PMC8024295; doi:10.1038/s41467-021-22256-3)
Supplement: Supplementary file 3 — Reporting summary [file 41467_2021_22256_MOESM3_ESM.pdf]

nature research

Corresponding author(s): Dr. Shouyang Wang
Dr. Dabo Guan

Last updated by author(s): Feb 25, 2021

Reporting Summary

Nature Research wishes to improve the reproducibility of the work that we publish. This form provides structure for consistency and transparency in reporting. For further information on Nature Research policies, see our Editorial Policies and the Editorial Policy Checklist.

Please do not complete any field with "not applicable" or n/a. Refer to the help text for what to use if an item is not relevant to your study.
For final submission: please carefully check your responses for accuracy; you will not be able to make changes later.

Statistics

For all statistical analyses, confirm that the following items are present in the figure legend, table legend, main text, or Methods section.

n/a	Confirmed	
☐	☒	The exact sample size (*n*) for each experimental group/condition, given as a discrete number and unit of measurement
☐	☒	A statement on whether measurements were taken from distinct samples or whether the same sample was measured repeatedly
☒	☐	The statistical test(s) used AND whether they are one- or two-sided<br>*Only common tests should be described solely by name; describe more complex techniques in the Methods section.*
☐	☒	A description of all covariates tested
☐	☒	A description of any assumptions or corrections, such as tests of normality and adjustment for multiple comparisons
☐	☒	A full description of the statistical parameters including central tendency (e.g. means) or other basic estimates (e.g. regression coefficient) AND variation (e.g. standard deviation) or associated estimates of uncertainty (e.g. confidence intervals)
☒	☐	For null hypothesis testing, the test statistic (e.g. *F*, *t*, *r*) with confidence intervals, effect sizes, degrees of freedom and *P* value noted<br>*Give P values as exact values whenever suitable.*
☒	☐	For Bayesian analysis, information on the choice of priors and Markov chain Monte Carlo settings
☐	☒	For hierarchical and complex designs, identification of the appropriate level for tests and full reporting of outcomes
☐	☒	Estimates of effect sizes (e.g. Cohen's *d*, Pearson's *r*), indicating how they were calculated

Our web collection on statistics for biologists contains articles on many of the points above.

Software and code

Policy information about availability of computer code

Data collection	n/a
Data analysis	Analyses and simulation are performed in Vensim software (PLE version 8.2.1).Regressions and statistical tests are performed using Stata software (Version 14.1)

For manuscripts utilizing custom algorithms or software that are central to the research but not yet described in published literature, software must be made available to editors and reviewers. We strongly encourage code deposition in a community repository (e.g. GitHub). See the Nature Research guidelines for submitting code & software for further information.

Data

Policy information about availability of data

All manuscripts must include a data availability statement. This statement should provide the following information, where applicable:
- Accession codes, unique identifiers, or web links for publicly available datasets
- A list of figures that have associated raw data
- A description of any restrictions on data availability

All original datasets used and the generated data from the results of the study are available at CEADS database (https://www.ceads.net/user/download-anonymous.php?id=1083). All data are also available from the corresponding author upon reasonable request.

Field-specific reporting

Please select the one below that is the best fit for your research. If you are not sure, read the appropriate sections before making your selection.

☐ Life sciences ☐ Behavioural & social sciences ☒ Ecological, evolutionary & environmental sciences

Ecological, evolutionary & environmental sciences study design

All studies must disclose on these points even when the disclosure is negative.

Study description	Quantify the current and estimate future energy consumption and the associated carbon emission output of Bitcoin blockchain operations in China.
Research sample	The research sample data contains the time-related Bitcoin blockchain time-series data including network hash rate, block size, transaction fee and difficulty that spans from January 1st, 2014 to January 1st, 2020. The average energy cost and carbon taxation in China are collected from the World Bank (data.worldbank.org). The site proportion of Bitcoin miners in China are set based on the regional statistics of Bitcoin mining pools collected from www.btc.com.
Sampling strategy	We extracted all the Bitcoin blockchain time-series data, including price, network hash rate, block size, transaction fee and difficulty, that were available up to the time of the study, which spans from January 1st, 2014 to January 1st, 2020.
Data collection	The Bitcoin time-series data was collected from www.btc.com. The data on average energy cost and carbon taxation in China are collected from the World Bank (data.worldbank.org). The site proportion of Bitcoin miners in China are set based on the regional statistics of Bitcoin mining pools collected from www.btc.com. All data mentioned above are collected by Shangrong Jiang and Yuze Li using MacBook Pro (13-inch, 2017, Four Thunderbolt 3 Ports).
Timing and spatial scale	The Bitcoin time-series data are monthly, global-wide data spanning from January 1st, 2014 to January 1st, 2020.
Data exclusions	No data were excluded.
Reproducibility	All 3 attempts to repeat the experiment were successful.
Randomization	No randomization was performed since all data were required for model development.
Blinding	No blinding was performed since outcome data were required for model development.

Did the study involve field work? ☐ Yes ☒ No

Reporting for specific materials, systems and methods

We require information from authors about some types of materials, experimental systems and methods used in many studies. Here, indicate whether each material, system or method listed is relevant to your study. If you are not sure if a list item applies to your research, read the appropriate section before selecting a response.

Materials & experimental systems

n/a	Involved in the study
☒	☐ Antibodies
☒	☐ Eukaryotic cell lines
☒	☐ Palaeontology and archaeology
☒	☐ Animals and other organisms
☒	☐ Human research participants
☒	☐ Clinical data
☒	☐ Dual use research of concern

Methods

n/a	Involved in the study
☒	☐ ChIP-seq
☒	☐ Flow cytometry
☒	☐ MRI-based neuroimaging

This checklist template is licensed under a Creative Commons Attribution 4.0 International License, which permits use, sharing, adaptation, distribution and reproduction in any medium or format, as long as you give appropriate credit to the original author(s) and the source, provide a link to the Creative Commons license, and indicate if changes were made. The images or other third party material in this article are included in the article's Creative Commons license, unless indicated otherwise in a credit line to the material. If material is not included in the article's Creative Commons license and your intended use is not permitted by statutory regulation or exceeds the permitted use, you will need to obtain permission directly from the copyright holder. To view a copy of this license, visit http://creativecommons.org/licenses/by/4.0/
